# Icariin Against Neurodegeneration: A Focus in Cell Death Pathways

**DOI:** 10.3390/ijms27052247

**Published:** 2026-02-27

**Authors:** Vinícius Rodrigues-Soares, Heitor Roque da Cruz, Gabriel Ferreira dos Santos, João Gabriel Restier, Pedro Castilho, Natan Giovanni Ferreira dos Santos, Elias Avgerino dos Santos, Daniel Souza Monteiro de Araújo, Karin da Costa Calaza, Rafael Brito

**Affiliations:** 1 Laboratory of Neuronal Physiology and Pathology, Department of Molecular and Cellular Biology, Institute of Biology, Fluminense Federal University, Niterói 24210240, RJ, Brazil; rodrigues_vinicius@usp.br (V.R.-S.); heitorcruz@id.uff.br (H.R.d.C.); ga_ferreira_santos@id.uff.br (G.F.d.S.); joao.restier@bioqmed.ufrj.br (J.G.R.); santoselias@id.uff.br (E.A.d.S.); rafaelbrito@id.uff.br (R.B.); 2 Graduate Program of Neurosciences, Institute of Biology, Fluminense Federal University, Niterói 24210201, RJ, Brazil; daniel.souzamonteirodearaujo@gmail.com; 3 Graduate Program of Biomedical Sciences, Biomedical Institute, Fluminense Federal University, Niterói 24210201, RJ, Brazil; 4 Laboratory of Retinal Neurobiology, Department of Neurobiology, Institute of Biology, Fluminense Federal University, Niterói 24210201, RJ, Brazil

**Keywords:** icariin, natural products, neurodegeneration, cell death

## Abstract

Distinct molecular pathways of cell death drive the progression of neurological pathologies. Targeting the signaling cascades induced by oxidative stress and inflammation within these pathways is central to developing cytoprotective therapies against apoptosis, ferroptosis, and excitotoxicity. In recent years, the flavonoid icariin has emerged as a promising candidate, showing significant potential to intercept these specific cell death mechanisms. However, while the current literature highlights icariin’s general neuroprotective effects, evidence regarding its precise interaction with distinct cell death modalities remains fragmented. This review synthesizes current knowledge to clarify the molecular logic by which icariin modulates these specific pathways in neurological models.

## 1. Introduction

The aging of the population has been accompanied by a rising incidence of age-related and metabolic disorders, such as Parkinson’s disease, Alzheimer’s disease, age-related macular degeneration, and type 2 diabetes [1]. These conditions place a substantial economic burden on health care systems and highlight the urgent need for innovative therapeutic strategies focused not only on treatment but also on prevention [2,3]. However, the therapeutic management of neurodegenerative disorders remains a critical pharmacological challenge, largely due to the multifactorial nature of neuronal cell death. Effective intervention requires agents capable not only of crossing the blood–brain barrier but also of simultaneously modulating distinct deleterious signaling cascades that drive disease progression.

Neuronal loss and the cognitive deficits associated with neurodegenerative pathologies involve events related to inflammation, oxidative stress, and apoptosis [4,5,6,7,8]. Aberrant redox signaling in oxidative stress damages organelles, proteins, lipids, and DNA; this cellular damage is consistent with post-mortem findings in patients with Alzheimer’s, Parkinson’s and Huntington’s disease [9]. Oxidative stress can trigger specific cell death pathways, including apoptosis and ferroptosis. In apoptosis, mitochondrial membrane damage leads to a decrease in membrane potential, promoting the intrinsic cascade, a known marker in major neurodegenerative diseases such as Alzheimer, Parkinson, and amyotrophic lateral sclerosis [10]. Conversely, ferroptosis is driven by hydroperoxides initiating chain reactions facilitated by transition metals like iron. This results in the generation of highly reactive lipid radicals, a process closely linked to Parkinson’s disease [11]. On the other hand, neuroinflammation creates an environment favorable for cell death [12]. The inflammatory response varies according to pathology, ranging from microglial and glial activation in Alzheimer’s disease to leukocyte migration in stroke or amyotrophic lateral sclerosis [13]. Chronic glial activation can drive both oxidative stress and inflammatory cell death, such as pyroptosis [13]. Other signaling pathways, such as endoplasmic reticulum stress, also promote neurodegeneration and interplay with apoptosis to drive Parkinson’s and Alzheimer’s disease progression [14,15]. Moreover, lysosomal dysfunction impairs the autophagy-dependent recycling of macronutrients and organelles, further contributing to neuronal death in multiple neurodegenerative diseases [16,17]. In this context, prenylated flavonoids have emerged as privileged scaffolds for neurodrug discovery due to their favorable lipophilicity and multimodal activity [18].

**Figure 1:**
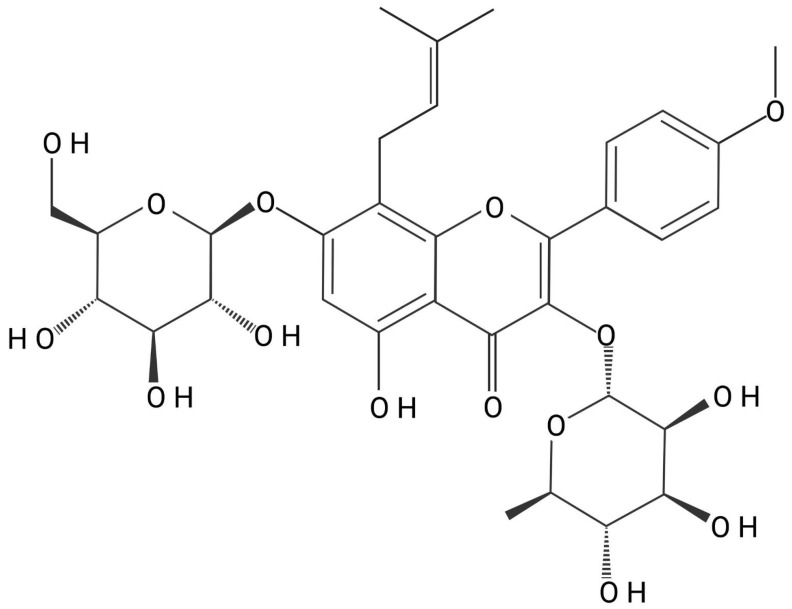
Molecule of Icariin (ICA).

Traditional medicine provides a valuable repository of bioactive compounds derived from herbs and roots that have been used for millennia to treat a wide variety of diseases. A well-known example is *Herba Epimedii*, widely used in traditional Chinese medicine for treating headaches, muscle weakness, and contracture in countries such as China, Korea, and Japan [19]. Icariin (ICA, C33H40O15) (Figure 1), the major bioactive constituent of *Herba Epimedii* (*Epimedium* spp.), represents a prime flavonoid candidate [20]. ICA, first identified in 1990 through chromatographic and spectroscopic methods, belongs to the class of prenylated flavonoids [21]. Despite its traditional use, the clinical translation of ICA in Western markets faces significant regulatory hurdles. A recent analysis of the European market highlights that *Epimedium* species are frequently found in products lacking proper authorization [22]. Unlike widespread botanicals such as Ginseng, *Epimedium* is currently absent from many recommendation documents worldwide, confining it to a borderline status between food supplement and medicine. To bridge the gap from an unregulated botanical to a targeted therapeutic, a precise understanding of its molecular mechanisms could facilitate translation [22].

Preclinical studies have shown that ICA exerts a wide spectrum of biological effects, including antioxidant, antitumor, anti-inflammatory, and protective activities [23]. In the central nervous system (CNS), ICA has been reported to counteract neuronal apoptosis, attenuate cognitive decline in experimental models of Parkinson’s and Alzheimer’s disease [24,25], and protect against diverse neuronal insults [24]. These beneficial effects are associated with its polyphenolic nature, which enables redox regulation by balancing reactive oxygen (ROS) and reactive nitrogen species (RNS) [24,26]. Beyond its antioxidant properties, recent evidence suggests that ICA’s neuroprotective potential also involves the release of neurotrophic factors [27,28], activation of pro-survival signaling pathways [24,29], and modulation of sirtuin family proteins, key regulators of cellular metabolism and stress response [30,31,32]. Moreover, ICA has been reported to inhibit apoptosis by reducing endoplasmic reticulum stress [24,31,33], suppressing glial reactivity [24,34], and restoring autophagic activity under pathological conditions [24,35]. Importantly, it can also interfere with ferroptosis, an iron-dependent and caspase-independent mode of regulated cell death [36].

The effects observed with ICA are consistent with findings reported for other flavonoids. Prunin, for example, has been shown to modulate several cellular pathways, presenting differently in healthy versus cancerous cells [37]. Many flavonoids can alleviate inflammation and oxidative stress, as well as activate apoptotic processes in tumors (anti-cancerous) while inhibiting it in healthy cells [38,39,40]. Although the anti-inflammatory mechanisms of polyphenolic flavonoids are not completely elucidated, their activity has been correlated with the inhibition of inflammatory signaling and reductions in proinflammatory mediators [41,42]. The antioxidative effects of flavonoids, on the other hand, have been linked to their role as ROS scavengers, lowered mitochondrial ROS production, and effective activation of nuclear factor erythroid 2-related factor 2 (Nrf2) [43,44,45,46]. As natural flavonoids, the actions of ICA are expected to present fewer negative side effects [37,47,48]. Taken together, these findings highlight ICA as a promising natural compound with multimodal actions in the CNS. This review synthesizes fragmented preclinical evidence to map these specific molecular targets (Supplementary Materials), providing the mechanistic validation necessary to support its future therapeutic standardization.

### Pharmacokinetics of Icariin

The pharmacokinetic profile of ICA represents a critical determinant of its biological activity and constitutes a major source of variability across experimental models. Importantly, pharmacokinetic constraints impose limitations on the interpretation of ICA’s neuroprotective effects, particularly in studies addressing CNS outcomes. ICA exhibits low oral bioavailability due to its diglycosylated structure, which limits direct intestinal absorption [20,49,50]. Following oral administration, ICA undergoes rapid systemic clearance, with reported half-lives ranging from approximately 1 to 3 h depending on the route of administration [20,49,50]. Intravenous delivery results in a rapid decline in plasma concentrations, with a half-life of approximately 0.5 h [51], whereas intragastric administration yields bioavailability values close to 12% and a half-life of approximately 1 h [52,53]. These data consistently indicate that ICA is rapidly eliminated from systemic circulation.

Tissue distribution studies indicate that ICA accumulates preferentially in peripheral organs such as the liver, lung, spleen, heart, kidney, brain and reproductive tissues, while its direct penetration into brain tissue appears limited [50]. This restricted CNS exposure suggests that the neuroprotective effects observed in preclinical models may not solely depend on high concentrations of the parent compound within brain parenchyma. Instead, these effects are likely shaped by the contribution of bioactive metabolites, indirect modulation of peripheral immune and inflammatory signaling, and cumulative low-level exposure capable of influencing glial and redox-sensitive pathways in the CNS.

A defining feature of ICA pharmacokinetics is its extensive intestinal and hepatic metabolism. Following oral administration, ICA is predominantly converted into bioactive metabolites, including icariside I, icariside II, and icaritin, through the combined actions of intestinal enzymes, gut microbiota β-glucosidases, and efflux transporters such as P-glycoprotein [49,54,55,56,57]. Notably, the intestine represents the primary site of ICA biotransformation, with approximately 90% of the orally administered compound metabolized prior to systemic circulation, whereas intravenous administration results in minimal conversion [58,59]. These metabolites can subsequently undergo conjugation reactions, generating glucuronidated derivatives such as icaritin-7-O-glucuronide and icaritin-3-O-rhamnoside-7-O-glucuronide, which may exhibit distinct pharmacodynamic properties [57]. This metabolic complexity has important implications for the interpretation of experimental findings. The reported neuroprotective effects attributed to ICA may reflect, at least in part, the actions of its metabolites rather than the parent molecule itself. Moreover, differences in dose, route of administration, formulation, and treatment duration across studies likely represent compensatory strategies to overcome rapid clearance and limited brain distribution, contributing to heterogeneity in the molecular pathways reported to be modulated by ICA.

Several formulation-based approaches have been explored to improve ICA bioavailability, including complexation with hydroxypropyl-β-cyclodextrin and liposomal encapsulation in propylene glycol systems [54,60]. These strategies increase systemic exposure, tissue distribution, and area under the curve, nearly doubling peak plasma concentrations in some models [60]. However, improvements in brain penetration remain modest, with increases of approximately 2%, and clearance rates largely unchanged [54,60]. These findings reinforce the notion that ICA-mediated CNS effects may involve indirect mechanisms, such as modulation of peripheral inflammation, systemic oxidative stress, or sustained low-level exposure rather than acute high brain concentrations.

While pharmacokinetic data consistently indicate rapid degradation and low oral bioavailability of ICA [50], the definition of an effective dose is also dependent on the target tissue and its associated biological barriers. Recent studies reveal a marked dose–response stratification between central and non-central targets, underscoring the impact of tissue distribution on pharmacological efficacy. For example, neuroprotection in models involving CNS dysfunction required relatively high oral doses (up to 100 mg/kg), as reported in models of encephalopathy-associated neural injury [61], whereas significant cytoprotection in ocular tissues, such as prevention of lens opacification, was achieved at substantially lower doses (20 mg/kg) [62]. This discrepancy suggests that, although ICA exerts potent biological effects at low systemic concentrations, achieving efficacy in CNS-related models likely requires higher doses, reflecting limitations in neuronal tissue exposure rather than intrinsic pharmacodynamic inefficacy. Accordingly, variability in effective doses across experimental models should be interpreted in light of pharmacokinetic constraints, including clearance rate, metabolic conversion, and tissue accessibility, rather than as inconsistencies in molecular mechanisms.

Altogether, pharmacokinetic data of ICA reveal rapid degradation, low oral bioavailability, and limited distribution to neural tissues [50]. Nevertheless, numerous studies employing oral administration of ICA in animal models consistently demonstrate neuroprotective effects, including the attenuation of inflammation [34,63], oxidative stress [64,65], and apoptosis [66,67], suggesting that effective biological activity can be achieved despite pharmacokinetic constraints when appropriate dose regimens are applied.

## 2. Anti-Inflammatory Actions of ICA

Inflammation is a fundamental biological response of the immune system to harmful stimuli such as pathogens, damaged cells, or irritants. In its acute form, it is a short-lived, localized process essential for defense and tissue repair, allowing for the elimination of the cause of injury and initiating recovery [68]. However, dysregulated or persistent inflammation contributes to disease onset and progression, including neurodegenerative disorders, by disrupting cytokine signaling, intracellular pathways, and glial homeostasis [68]. The inflammatory response can also originate from the nervous tissue itself, a response of glial cells in pathological conditions that is called neuroinflammation. Understanding the molecular and cellular mechanisms that govern inflammatory response is therefore crucial for developing therapeutic strategies against neurodegeneration and restoring homeostasis.

ICA has shown promising therapeutic potential in neurodegenerative conditions, particularly in settings where immune-mediated inflammation is a key driver of progressive neuronal and glial degeneration. Wei and coworkers (2016) demonstrated that ICA exerts estrogen-like effects in experimental autoimmune encephalomyelitis, a chronic demyelinating inflammatory disease that promotes neurodegeneration in multiple sclerosis, through modulation of estrogen receptor alpha and beta (ERα/ERβ) expression, contributing to preservation of cerebral white matter and attenuation of secondary neuronal damage [69]. At the level of the central nervous system, Cong et al. (2020) reported that ICA suppresses microglial reactivity and downregulates proinflammatory mediators, including tumor necrosis factor alpha (TNF-α) and inducible nitric oxide synthase (iNOS), through inhibition of nuclear factor kappa-light-chain-enhancer of activated B-cells (NF-κB) signaling, further limiting inflammation-driven neurodegeneration in encephalomyelitis [70]. Supporting these findings, Wang et al. (2022) demonstrated that ICA attenuates glial-mediated inflammation in a C57BL/6 mouse model of autoimmune uveitis, a condition capable of inducing secondary neurodegenerative changes in central nervous system structures such as the retina [71]. In this context, ICA shifted microglial activity toward a less reactive, tissue-protective profile via peroxiredoxin-3 (PRDX3), a mitochondrial enzyme involved in hydrogen peroxide (H_2_O_2_) detoxification, whose silencing abolished ICA’s anti-inflammatory effects [71]. Consistently, Shen et al. (2015) showed that ICA induces systemic immune modulation in the same model by suppressing T helper 1 (Th1) and T helper 17 (Th17) differentiation, which was associated with reduced blood–brain barrier (BBB) permeability and improved clinical outcomes [72]. Corroborating with these findings, Pokkula and Thakur (2021) demonstrated that oral ICA administration in Wistar rats subjected to a sciatic nerve injury reduced proinflammatory cytokines, Interleucin-6 (IL-6) and TNF-α, in sciatic nerve homogenates [73]. These molecular changes were accompanied by a significant reduction in pain scores. Further, ICA-loaded nanofibers reduced inflammation markers (TNF-α, Hypoxia-Inducible Factor 1 Alpha [HIF-1α], IL-6, and Interleukin 1-β [IL-1β]) in rat sciatic nerve homogenates [74].

In models of cognitive impairment and neurodegenerative disease, ICA consistently suppresses inflammatory pathways that exacerbate neuronal loss and synaptic dysfunction. In postoperative cognitive dysfunction and Alzheimer’s disease models, ICA improves cognitive performance by reducing hippocampal inflammation, inhibiting microglial activation, and limiting neuronal injury through suppression of Toll-like receptor 4 (TLR4)/NF-κB signaling [75]. Additionally, ICA inhibits cGAS–STING signaling and its downstream effectors TANK-binding kinase 1 (TBK1) and interferon regulatory factor 3 (IRF3), thereby reducing type I interferon production and chronic inflammatory amplification, processes closely linked to neurodegenerative progression in Alzheimer’s disease [76]. ICA further limits disease pathology by reducing β-amyloid plaque accumulation, transforming growth factor beta 1 (TGF-β1) expression, ceramide levels, and microglial pyroptosis via downregulation of the cyclooxygenase-2 (COX-2)–NLRP3 inflammasome–gasdermin D axis [75,76,77,78]. A similar mechanistic link between inflammation and neurodegeneration is evident in epilepsy, where recurrent seizures promote excitotoxicity, oxidative stress, and chronic microglial activation. In this context, ICA reduces seizure severity and mortality during the acute phase and improves cognitive performance during the chronic phase by promoting microglial polarization toward an anti-inflammatory phenotype, thereby limiting inflammation-associated neurodegenerative damage [79]. ICA also exerts robust neuroprotective effects in ischemic and vascular-related neurodegeneration. In cerebral ischemia and vascular dementia models, where hypoxia-induced inflammation and oxidative stress drive progressive neuronal loss, ICA improves neurological outcomes, reduces cerebral edema, and suppresses microglial activation and proinflammatory cytokine production [34,80]. These anti-inflammatory effects are accompanied by restoration of synaptic and neurotrophic signaling, including increased brain-derived neurotrophic factor (BDNF), tropomyosin receptor kinase B, extracellular signal-regulated kinases (ERK), cAMP response element-binding protein (CREB), synaptophysin, postsynaptic density protein 95 (PSD-95), glutamate ionotropic receptor NMDA type subunit 2B (GluN2B), and calcium/calmodulin-dependent protein kinase II (CaMKII) expression, supporting neuronal survival and synaptic plasticity in degenerative contexts [81].

**Figure 2:**
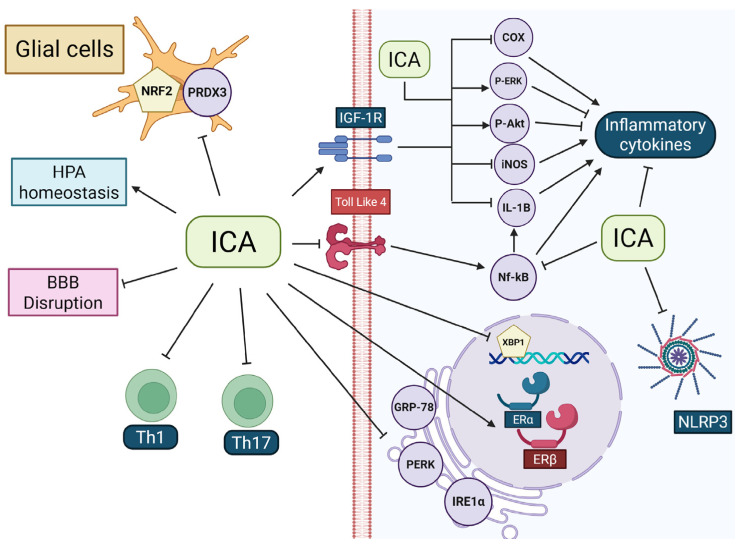
ICA’s effect on the inflammatory response. ICA exerts both local and systemic anti-inflammatory effects, reducing inflammatory cytokines, regulating the hypothalamic–pituitary–adrenal axis (HPA), regulating blood–brain barrier (BBB) disruption, attenuating Th1 and Th17 responses and microglial activation in a peroxiredoxin 3 (PRDX3) and Nrf2-dependent manner. At the molecular level, ICA reduces the production of inflammatory cytokines and promotes the activation of IGF-1R receptors, leading to decreased expression of nuclear factor kβ (Nf-kβ), inducible nitric oxide synthase (iNOS), cyclooxygenases (COX) and Interleukin 1-β (IL-1β), while increasing the phosphorylation of extracellular signaling-regulated kinases (ERKs) and protein kinase B (Akt), thereby contributing to the attenuation of the inflammatory response. In addition, ICA has been shown to mediate other inflammatory responses by reducing the formation of NOD-like receptor protein 3 (NLRP3), expression of X-box binding protein 1 (XBP1), and activation of Glucose-Regulated Protein 78 (GRP-78), protein kinase RNA-like endoplasmic reticulum kinase (PERK), and inositol-requiring enzyme 1 alpha (IRE1α). These effects have also been associated with the modulation of estrogen receptor α and β (ERα and ERβ) receptors, contributing to reduced inflammatory demyelination.

The anti-inflammatory and neuroprotective effects of ICA are, in several contexts, mediated by activation of nuclear factor erythroid 2–related factor 2 (Nrf2), a transcription factor critically involved in cellular survival and redox homeostasis. In BV-2 microglial cells, ICA suppressed lipopolysaccharide-induced production of proinflammatory mediators and glial activation in an Nrf2-dependent manner, while pharmacological inhibition of heme oxygenase 1 (HO-1) abolished these effects, highlighting the central role of the Nrf2/HO-1 axis [82]. Consistently, ICA reduced dopaminergic neurotoxicity and glial-driven neuroinflammation in a 6-hydroxydopamine-induced Parkinson’s disease model through Nrf2-dependent mechanisms [83], and similar anti-inflammatory and functional benefits were observed in models of spinal cord injury, where ICA treatment correlated with significant motor recovery [64,84]. Mechanistically, accumulating evidence indicates that ICA-mediated Nrf2 activation is regulated upstream by sirtuin 1 (SIRT1). In an amyotrophic lateral sclerosis model, inhibition of SIRT1 abolished ICA-induced Nrf2 activation and reversed motor improvements, demonstrating a functional dependence on the SIRT1/Nrf2 axis [85]. Supporting this hierarchical relationship, independent studies showed that pharmacological or genetic disruption of SIRT1 or Nrf2 signaling negated neuroprotective effects elicited by other compounds, confirming SIRT1 as an upstream regulator of Nrf2-mediated cytoprotective responses [86,87]. Notably, ICA does not alter Nrf2 mRNA expression but increases nuclear Nrf2 protein levels [82,83], indicating a post-transcriptional mechanism involving enhanced protein stability and nuclear translocation. Collectively, these findings support a model in which ICA attenuates glial-driven neuroinflammation and neurodegeneration by modulating the SIRT1/Nrf2/HO-1 axis, thereby restoring the balance between antioxidant and proinflammatory signaling pathways, including crosstalk with NF-κB, and highlighting this pathway as a promising therapeutic target in neuroinflammation-driven neurodegenerative diseases (Figure 2) [87].

In addition to the SIRT1/Nrf2/HO-1 axis, ICA also engages insulin-like growth factor 1 receptor (IGF-1R)–dependent signaling to limit neuroinflammation and excitotoxic damage. Icariin and icaritin suppressed lipopolysaccharide-induced expression of TNF-α, IL-1β, cyclooxygenase-2 (COX-2), and inducible nitric oxide synthase (iNOS), while enhancing basal phosphorylation of ERK1/2 and protein kinase B (AKT) [88]. Concomitantly, ICA upregulated the astrocytic glutamate transporters GLT-1 and GLAST, supporting improved glutamate clearance and neuronal protection. Importantly, all anti-inflammatory and regulatory effects were abolished by pharmacological IGF-1R inhibition, confirming IGF-1R–dependent signaling [88].

Taken together, these findings demonstrate that ICA targets neurodegeneration by converging on key inflammatory and oxidative pathways that link chronic glial activation to neuronal dysfunction. By suppressing proinflammatory signaling cascades such as NF-κB, cGAS–STING, and NLRP3 inflammasome activation, while simultaneously enhancing SIRT1/Nrf2/HO-1–mediated cytoprotective responses, ICA restores the balance between inflammatory and antioxidant signaling, preserving neuronal integrity and function in neurodegenerative conditions driven by sustained neuroinflammation [34,61,70,71,72,73,75,76,77,80,88,89]. Table 1 shows information from all the studies related to this topic.

## 3. ICA as a Potent Antioxidant Agent

Multiple animal and cellular models of nervous system disorders consistently demonstrate the disruption of redox signaling as a pathological hallmark. Called as oxidative stress, this is defined as a homeostatic imbalance between oxidizing molecules and antioxidant defenses, where the prevalence of ROS and NOS, such as hydrogen peroxide and peroxynitrite, results in molecular damage [93,94]. Oxidative stress is also frequently associated with an enzymatic imbalance, in which the excessive production of radicals by enzymes such as such as Nicotinamide Adenine Dinucleotide Phosphate (NADPH) oxidase and the dysfunction of the mitochondrial respiratory chain outweigh the neutralization capacity of endogenous antioxidant enzymes. These include Superoxide Dismutase (SOD), Catalase (CAT), and Glutathione Peroxidase (GSH-Px) [93,95,96]. As a signaling antioxidant response, cell signals activate specific intracellular pathways, mainly mediated by the translocation of the transcription factor Nrf2 to the nucleus [97]. There, by binding to the Antioxidant Response Element, Nrf2 induces the expression of protective genes (such as HO-1 and NQO1) [98], restoring cellular balance and preventing regulated cell death pathways.

Importantly, a growing body of evidence from cellular and animal models of neurodegenerative disorders demonstrates that ICA effectively counteracts oxidative stress-induced neuronal injury through multiple, partially overlapping signaling pathways. Across diverse experimental systems, ICA decreases ROS production [26,64,65,78,82,83,84,85,89,91,99,100,101,102,103,104,105,106,107,108,109,110,111,112,113,114,115,116] and lipid peroxidation (malondialdehyde, MDA) [64,78,84,91,101,108,110,116]; preserves endogenous antioxidant defenses, including GSH [65,106,110,116], SOD [65,78,108,110,111,116], CAT [78,107], and GSH-Px [110,116]; and stabilizes mitochondrial function [65,84,108,111,115]. In addition, ICA modulates critical molecular nodes that connect oxidative stress to regulated forms of cell death, such as caspase-3 activation [26,78,84,101,106], p53 [106], glycogen synthase kinase-3 beta (GSK-3β)/Tubulin-associated unit (Tau) [111,113], sirtuins [65,107,116], and Nrf2 [82,83,105,116].

In vivo studies using senescence-accelerated mice (SAMP8) have shown that chronic ICA treatment enhances the activity of SOD and GSH-Px in brain tissue [111]. Similarly, in haloperidol-induced Parkinson’s models, ICA significantly increased the activity of glutathione S-transferase, CAT, and SOD in neuro-glial cells [108].It is important to note, however, that the efficacy of ICA may be context-dependent. For instance, in certain cerebral ischemia models, ICA monotherapy showed limited efficacy [104], whereas its combination with Panax notoginseng resulted in a synergistic reduction of oxidative stress markers [117]. This suggests that while ICA is a potent antioxidant modulator, its effectiveness can be influenced by the specific pathological environment or the presence of synergistic compounds.

Beyond systemic effects, in vitro studies suggest that ICA directly influences the antioxidant response of neurons. In PC12 cells treated with diabetic encephalopathy metabolites, ICA increased SOD activity and reduced MDA accumulation [109]. Similarly, in primary cortical neurons, ICA upregulated Prx1 mRNA and CAT activity via a SIRT1-dependent mechanism. Complementing these enzymatic responses, ICA prevents GSH depletion in PC12 cultures exposed to H2O2 by inhibiting the c-Jun N-terminal Kinase (JNK) and p38 Mitogen-Activated Protein Kinase (MAPK) pathways [107]. In spinal cord injury models, ICA’s antioxidant capacity was evidenced by its ability to prevent the reduction of GSH levels in neural tissue [64,84].

ICA also exerts neuroprotection by directly stabilizing mitochondrial function and preventing mitochondria-dependent apoptosis. In models of spinal cord injury and Sodium Azide (NaN3)-induced stress, ICA restored Mitochondrial Membrane Potential and enhanced ATP production [84,112]. ICA helps maintain mitochondrial homeostasis by inhibiting the formation of the mitochondrial permeability transition pore. Furthermore, ICA acts as an antagonist of alpha-amino-3-hydroxy-5-methyl-4-isoxazolepropionic acid (AMPA) receptors, specifically reducing excessive calcium influx [101]. By preventing this calcium overload, ICA averts the subsequent breakdown of Ca^2+^-dependent regulatory processes that typically triggers mitochondrial collapse. Corroborating this evidence, in APP/PS1 transgenic rats, ICA reduced intracellular iron accumulation, thereby limiting iron-catalyzed ROS generation (Fenton reaction) that typically damages mitochondrial membranes [115].

However, ICA can act as a sophisticated signaling modulator in the oxidative stress response. The most prominent signaling mechanism for ICA is the activation of the Nrf2 cascade. In different neurodegeneration models, ICA promoted Nrf2 nuclear translocation, leading to the expression of HO-1 and NQO1 [82,83,106]. This effect was notably absent in Nrf2-knockout models, where ICA’s protective effects were largely abolished. Another study related ICA action against neurotoxicity through the SIRT3/PGC-1α signaling axis [118], demonstrating SIRT-3 as an essential signaling axis for maintaining the GSH/GSSG ratio and reducing oxidative damage to mitochondrial membranes by PGC-1α. In rotenone-induced Parkinson’s models, ICA prevented the suppression of Sirtuin 3 (SIRT3) and Peroxisome proliferator-activated receptor-gamma coactivator 1-alpha PGC-1α [65,82].

ICA activates the PI3K/Akt/GSK-3β survival pathway to protect neurons from NaN3)-induced apoptosis [112]. By inhibiting the c-Jun N-Terminal Kinase (JNK) and p38 MAPK pathways, ICA prevents DNA oxidation and subsequent cell death [107]. Reinforcing these findings, computational modeling reveals that ICA has a high binding affinity for targets associated with Brain-Derived Neurotrophic Factor (BDNF) signaling. This interaction likely enhances neuronal survival and plasticity, while simultaneously inhibiting Receptor for Advanced Glycation Endproducts (RAGE) and Glutamate Ionotropic Receptor AMPA Type Subunit 1 (GRIA1), both of which are central to redox deregulation [110,113].

**Figure 3:**
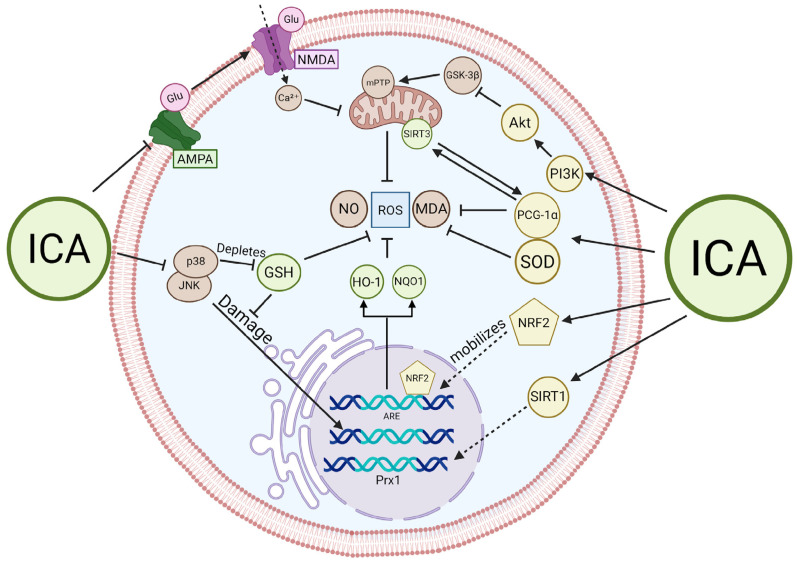
ICA’s antioxidant effects. ICA regulates oxidative stress by acting on multiple targets, thereby ameliorating damage caused by oxidative insults such as hypoxia/reperfusion, H_2_O_2_, and NaN_3_. Furthermore, ICA activates various pathways that maintain mitochondrial homeostasis, such as the phosphoinositide 3 kinase (PI3K)/protein kinase B (Akt)/glycogen synthase kinase-3 beta (GSK3β) pathway, which inhibits the formation of the mitochondrial permeability transition pore (mPTP) and the subsequent influx of calcium to the mitochondria. ICA also inhibits calcium-related oxidative stress by targeting AMPA receptors, reducing NMDA receptor activation, and calcium influx. This prevents alterations in several calcium-dependent signaling pathways and supports the maintenance of mitochondrial function. Furthermore, ICA decreases the accumulation of oxidative markers such as malondialdehyde (MDA), nitric oxide (NO), and reactive oxygen species (ROS) by promoting the expression and efficiency of antioxidant factors, including PCG-1α, sirtuin 3 (SIRT3), superoxide dismutase (SOD), glutathione (GSH), HO-1, and NAD(P)H dehydrogenase 1 (NQO1), by promoting the translocation of NRF2 and sirtuin 1 (SIRT1) to the nucleus and the subsequent expression of the antioxidant response element (ARE)- and peroxiredoxin (Prx1)-modulated genes. Glutathione (GSH) levels are also maintained by the inhibition of the p38/JNK pathway, reducing DNA damage and subsequent apoptosis.

Interestingly, ICA’s neuroprotective profile extends significantly to the glial microenvironment. In models of neuroinflammation, ICA administration reduced microglial populations and inhibited the release of pro-inflammatory mediators. In LPS-stimulated BV-2 microglia, ICA bolstered antioxidant capacity by upregulating the Nrf2/HO-1 pathway, effectively suppressing the “cytokine storm” associated with oxidative stress [92]. Crucially, in cuprizone-induced demyelination models, ICA treatment was associated with reduced ROS levels and enhanced maturation of oligodendrocyte progenitor cells. This suggests that ICA may promote a redox-permissive environment essential for oligodendrocyte metabolic health and subsequent remyelination [92]. By mitigating oxidative stress within the glial microenvironment, ICA likely provides an indirect, secondary layer of neuroprotection through the preservation of axonal insulation [26,85] (Figure 3).

These comparative findings reveal a conserved molecular logic underlying ICA’s efficacy across distinct pathologies. While both neurons and glia utilize the Nrf2 pathway, the outcomes differ while in glia, Nrf2 activation primarily dampens inflammation and supports remyelination [85,92], in neurons, it directly inhibits apoptosis and restores redox signaling. The comprehensive neuroprotective action of ICA, incorporating SIRT1 regulation, mitochondrial stabilization, and glial modulation, positions it as a robust candidate for treating diverse CNS neurodegenerative disorders [119,120] (Table 2).

## 4. ICA as a Negative Regulator of Apoptosis

Apoptosis is a tightly regulated form of programmed cell death essential for tissue homeostasis; however, its dysregulation is a central driver of neurodegenerative pathology. While classically categorized into intrinsic (mitochondrial) and extrinsic (death receptor) pathways, these signaling cascades do not operate in isolation but instead function as an integrated network [122,123]. For example, both pathways converge on the activation of executioner caspases, such as caspase-3, which orchestrate the final dismantling of the cell, including DNA fragmentation [122,123]. Cells possess an adaptive “brake” system mediated by Inhibitors of Apoptosis (IAPs), which counterbalance these cell death triggers. As reviewed by Marivin et al. (2012), IAPs [such as X-Linked Inhibitor of Apoptosis Protein (XIAP) and Cellular Inhibitor of Apoptosis Proteins 1 and 2 (cIAP1/2)] provide a critical adaptive response to oxidative stress by directly binding to and inhibiting processed caspases (3, 7, and 9) [124]. This mechanism allows neurons to tolerate transient stress without committing to cell death. Consequently, neuroprotective strategies must not only block upstream triggers like oxidative stress or ER stress but also reinforce this adaptive IAP-mediated threshold [125,126].

Apoptotic cell death in the pathogenesis of Alzheimer’s disease is associated with the activation of pro-apoptotic mediators, such as caspase-3 and -6, Bcl-2-associated X protein (Bax), and p53 upregulated modulator of apoptosis (PUMA), while anti-apoptotic mechanisms, such as B-cell lymphoma 2 (Bcl-2), Survivin and B-cell lymphoma-extra-large (Bcl-xL), are suppressed [127]. Several signaling pathways contribute to this imbalance, most notably PI3K/Akt, JNK/MAPK, and mechanistic target of rapamycin (mTOR). For instance, oxidative stress and inflammation drive activation of the JNK/MAPK pathway, which promotes apoptotic responses [127]. In parallel, impairment of the PI3K/Akt survival pathway enhances neuronal vulnerability by activating GSK-3β, thereby facilitating an Aβ/JNK/p53-induced cascade that culminates in neurofibrillary tangles (NFT) formation [127].

ICA demonstrates robust neuroprotective potential in Alzheimer’s disease models by mitigating core pathological hallmarks through the modulation of integrated apoptotic pathways. In vitro evidence in PC12 cell lines and primary cortical neurons indicates that ICA blocks amyloid-beta Aβ-induced neurotoxicity and sodium azide-induced mitochondrial dysfunction, resulting in the restoration of glucose metabolism and the reduction of tau protein hyperphosphorylation via activation of the PI3K/Akt/GSK3β pathway [106,112,114,128,129] (Table 3). These cellular findings are consistent with results from multiple in vivo models, including APP/PS1, 3xTg-AD, Tg2576, and SAMP8 mice. In these models, chronic ICA administration reduces APP expression and insoluble Aβ1-40 and Aβ1-42 levels [29,67,130,131] (Table 3). Specifically in the SAMP8 model, treatment attenuates memory impairment and reduces Aβ1-42 levels through the downregulation of BACE1, alongside increasing Bcl-2 expression and reducing Bax levels [67] (Table 3). Collectively, these actions promote the suppression of endoplasmic reticulum stress-induced apoptosis and the restoration of the Bax/Bcl-2 ratio, thereby preserving neuronal density in the hippocampus [29,130,131] (Table 3).

In the context of Parkinson’s disease, ICA consistently preserves dopaminergic integrity across various preclinical models by targeting pro-apoptotic factors that drive neuronal loss in the substantia nigra [25,132,134]. Studies utilizing 6-OHDA, Haloperidol, and MPTP (1-methyl-4-phenyl-1,2,3,6-tetrahydropyridine) models demonstrate that ICA administration, either alone or combined with levodopa, mitigates the loss of tyrosine hydroxylase-positive neurons and attenuates dopamine depletion in the striatum [25,132,134] (Table 3). These effects translate into the restoration of motor performance and enhanced antioxidant capacity, processes mediated by the activation of PI3K/Akt and MEK/ERK signaling pathways, as well as the inhibition of GSK3β activity [25,132,134] (Table 3). In PC12 cells, ICA confers cytoprotection against 6-OHDA-induced toxicity by reducing the Bax/Bcl-2 ratio and decreasing the proportion of cells in early apoptosis [25] (Table 3).

Advanced glycation end products (AGEs) are related to the occurrence of diabetic encephalopathy, a major complication of diabetes mellitus. Neuron apoptosis is a mechanistic factor on cognitive decline shown in streptozotocin induced animal models [143]. In a vitro model PC12 cell exposed to advanced glycation end products, ICA-treated groups had fewer apoptotic neurons and an overall reduction in caspase 3 and 9 levels. ICA directly inhibited Bax translocation to mitochondria, a mechanism central to its neuroprotection. Furthermore, ICA attenuated mitochondrial depolarization and restored antioxidant cell capacity, reinforcing its role in oxidative stress and apoptosis regulation [109]. Additionally, in primary hippocampal neuronal cell cultures from neonatal Sprague Dawley rats, Liu et al. (2011) reported that ICA suppressed corticosterone-induced apoptosis [134]. This protective effect was associated with inhibition of p38/MAPK activation and prevention of mitochondrial dysfunction, including preservation of mitochondrial membrane potential and suppression of caspase-3 activity [134]. In a similar model, it was demonstrated in neuronal primary hypothalamic cell culture that ICA prevented corticosterone-induced cell death via activation of the PI3-K/Akt pathway [135].

Cavernous nerve crush is an established model of axonotmesis [144], in which Wallerian degeneration and apoptosis of nitrergic nerves occur [145,146]. Conversely, in 12-week-old rats with cavernous nerve injury treated orally with ICA showed no differences in apoptosis markers in penile tissue homogenates [136]. However, the ICA-treated group exhibited a higher number of nerve fibers stained for neural nitric oxide synthase (nNOS) and upregulated nNOS expression, suggesting that ICA could rescue nitrergic neurons [136]. Moreover, in Wistar rats subjected to partial sciatic nerve ligation, chronic oral ICA administration reduced Bax and Bcl-2 protein levels and was associated with significant attenuation of neuropathic pain. This type of pain is suggested to activate the apoptotic pathway; thus, the reduction in these proteins could represent a greater protection of the sciatic nerve [73].

ICA demonstrated a robust neuroprotective effect against ischemic injury. Across diverse in vitro models, including oxygen–glucose deprivation/reperfusion (OGD/R) in neuronal cell lines (N2a [93] and PC12 [91,141]) and in primary cortical neurons [31,101,138]. In agreement, ICA benefits are also conserved in neonatal hypoxic–ischemic brain damage and adult middle cerebral artery occlusion in vivo models [138,139,140]. Treatment significantly improved cell viability by directly suppressing apoptotic pathways, as evidenced by dose-dependent reductions in cleaved caspase-3 and Bax expression, together with increased Bcl-2 levels [93,140,141]. Mechanistically activating pro-survival pathways while simultaneously suppressing proinflammatory and pro-apoptotic pathways (Table 3) [34,66,93,138]. In addition, ICA promotes cytoprotective autophagy through estrogen receptors (ERα/ERβ) activation and strengthens antioxidant defenses by upregulating Nrf2 and PPAR signaling [27]. ICA also exhibited a broader effects linked of endoplasmic reticulum stress pathways and pyruvate kinase M2 (PKM2)-dependent signaling [80,138], a conserved antioxidant action reducing ROS production [66,101,140,141], restored cytosolic Ca^2+^ homeostasis [101], and mitigated neuroinflammation [34,66,80,91,93,138] through inhibition of microglial activation, thereby preventing multiple apoptotic triggers [80,138]. These molecular changes translate into improved functional outcomes, including reduced infarct volume, diminished cerebral edema, and enhanced neurological performance.

**Figure 4:**
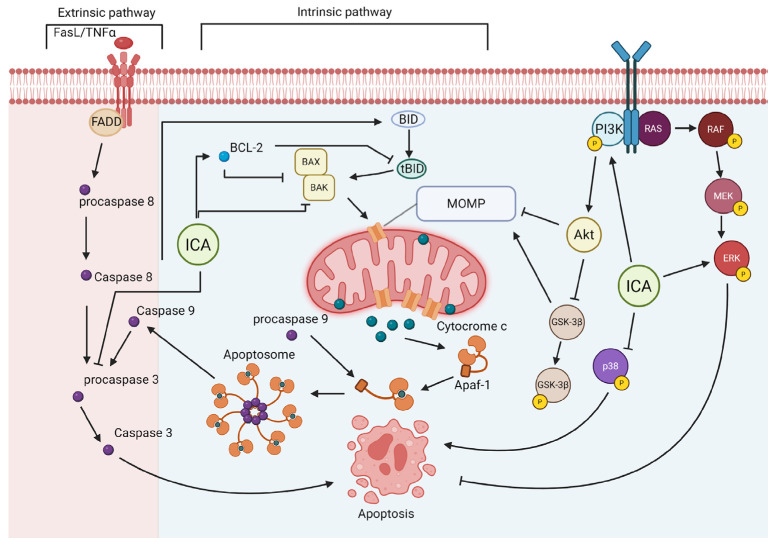
The influence of ICA on the apoptotic process. The extrinsic pathway is initiated by death receptors located on the plasma membrane. These receptors activate caspase-8, which subsequently activates caspase-3, culminating in apoptosis. In the intrinsic pathway, internal stimuli induce the oligomerization of BAX and BAK, which can be inhibited by ICA, and the Mitochondrial Outer Membrane Permeabilization (MOMP), which is inhibited by ICA’s influence over the PI3K/Akt/GSK-3β pathway. This oligomerization results in the release of cytochrome c into the cytosol, promoting apoptosome formation and the cleavage of caspase-9, which subsequently activates caspase-3 to execute apoptosis. Due to ICA’s inhibition of the oligomerization of BAX/BAK, the activation of caspases 9 and 3 is also inhibited, preventing apoptosis. The extrinsic pathway may also activate the intrinsic pathway through the cleavage of the Bid protein, generating a truncated form of this protein that triggers MOMP. Furthermore, ICA may regulate the BAX/Bcl ratio, activate the p-ERK pathway, and inhibit p-p38, thereby promoting cell survival.

Several apoptosis-related death models demonstrated that ICA can prevent neurodegeneration and also offer protection in non-degenerative neurological disorders such as depression [85], epilepsy [102] and schizophrenia [137]. ICA exhibits a robust multi-target neuroprotective profile mainly consistently targeting Bax/Bcl-2 axis and reducing caspase-3 activation across different pathological contexts (Figure 4). To mechanistically position ICA within the broader flavonoid signaling landscape, it is essential to synthesize its effects in contrast to the pro-apoptotic profile often reported in oncology. Recent comprehensive analyses describe a conserved pattern in neoplastic cells, where flavonoids such as ICA, quecetin [147], prunin [37] and isorhamnetin [119] induce apoptosis by inhibiting survival kinases, specifically the PI3K/Akt axis, suppressing NF-κB signaling and triggering mitochondrial dysfunction via reactive oxygen species accumulation. However, ICA exhibits a pathway-specific divergence in the CNS. Consistent with the neuroprotective signaling patterns reviewed for structurally related polyphenols, ICA sustains, rather than disrupts, PI3K/Akt and ERK1/2 phosphorylation in mature neuronal models [148]. This supports the hypothesis that flavonoid-driven modulation of redox and intrinsic apoptotic control is context-dependent: while these compounds exploit metabolic vulnerabilities to eliminate proliferating tumor cells, they reinforce intrinsic survival machinery in differentiated neurons. This duality highlights ICA as a promising agent for CNS preservation.

## 5. ICA and Autophagy

Autophagy is a lysosome-dependent degradation pathway essential for maintaining cellular homeostasis and can be classified into macroautophagy, microautophagy, and chaperone-mediated autophagy. Macroautophagy depends on the Unc-51-like Autophagy Activating Kinase 1–Autophagy-Related Genes (ULK1-ATG) complex and recruitment of the class III PI3K complex for initiation and elongation of the autophagosome membrane [149]. Macroautophagy represents autophagy per se and is responsible for the elimination of deleterious proteins and dysfunctional organelles, such as mitochondria. The selective removal of damaged mitochondria, a process known as mitophagy, promotes mitochondrial quality control and involves upregulation of PINK1 and Parkin expression [150], leading to the targeted elimination of dysfunctional mitochondria before they can release pro-apoptotic factors [150]. In contrast, during microautophagy, the lysosomal membrane undergoes invagination or protrusion to directly engulf cytoplasmic contents [149]. Chaperone-mediated autophagy (CMA) targets proteins containing a Lysosomal Targeting Motif (KFERQ), recognized by Heat Shock Cognate Protein 70 (HSC70) and translocated into the lysosomal lumen via the Lysosomal-Associated Membrane Protein 2A (LAMP2A) [151].

The autophagic response is activated in the presence of cellular damage as an adaptive mechanism for cell survival. However, its role in cell survival is ambiguous and associated with autophagic flux. While basal autophagy acts as a cytoprotective mechanism for recycling damaged organelles/dysfunctional proteins [150], excessive or dysregulated autophagy can drive “autophagy-dependent cell death” [150]. In this context, therapeutic agents must have more than a binary influence on autophagic responses. Current evidence indicates that ICA functions as a context-dependent modulator, exerting opposing effects depending on whether the pathological state involves excessive activation of maladaptive autophagy or functional impairment of adaptive autophagy [152]. The outcome between survival and death is largely governed by the interaction between the autophagy initiator Beclin-1 and the anti-apoptotic protein Bcl-2 [153]. Under basal conditions, Bcl-2 binds to Beclin-1, preventing the assembly of the PI3K-III complex and inhibiting autophagy initiation. In conditions of acute stress, such as oxygen–glucose deprivation/reperfusion, this balance is disrupted [141].

Conversely, in chronic neurodegenerative models characterized by the accumulation of misfolded proteins, ICA appears to stimulate autophagy to facilitate adaptive clearance. For example, upregulating p62 in mice brain via gut microbiota manipulation mitigated amyloid-β toxicity and improved cognitive function in mice by enhancing autophagic clearance [154]. With regard to ICA, treatment has been shown to improve neuronal morphology, cell viability, and behavioral test performance in mice, likely by reducing amyloid-β accumulation in APP/PS1 transgenic models and Aβ1-42-treated PC12 cells. However, the upregulation of autophagy- and mitophagy-related proteins was observed only when ICA was combined with ꞵ-Asarone [155]. Notably, in a prenatal stress depression model, ICA treatment reduced hippocampal cell death and improved depressive-like behavior via modulation of Sirt-1/PGC-1α mitochondrial dynamics. Concurrently, ICA promoted mitophagy by increasing mitochondrial PINK1 and Parkin expression, elevating the Microtubule-Associated Protein 1 Light Chain 3, form II and I (LC3II/LC3I) ratio, and reducing P62 levels [156]. Interestingly, in Aβ1–42-injected rats, increased LC3 turnover was not followed by a decrease in P62, suggesting impaired autophagic flux in this Alzheimer’s disease model. In this context, ICA treatment reduced LC3-II, Cathepsin D, and Beclin-1 levels, while enhancing Akt and phosphorylated ribosomal protein S6 kinase bet-1 p-p70S6K activation, a key autophagy regulator, thereby reestablishing autophagic homeostasis [157].

ROS, largely produced in mitochondria, are well-established inducers of autophagy [158]. Keap1 acts as a redox sensor and induces Nrf2 degradation via proteasomal and autophagic pathways, thereby enabling its nuclear translocation. Nrf2 promotes the transcription of antioxidant response element (ARE)-driven genes, including p62, which functions as a selective autophagy adaptor by binding both ubiquitinated proteins and LC3, thereby accelerating autophagic clearance [159]. In addition, several Atg proteins are susceptible to cystine oxidation, indicating that ROS can directly modulate autophagic machinery [160]. ROS may also activate autophagy indirectly through inhibition of the PI3K/Akt/mTOR pathway or activation of MAPK signaling cascades, including JNK, p38, and ERK [160]. Oxygen–glucose deprivation/reperfusion (OGD/R) is characterized by increased ROS production and cell death [161]. PC12 cells exposed to OGD (2 h) followed by 24 h of reperfusion, increased apoptosis, and upregulation of the autophagy markers Beclin-1 and LC3-II were observed, suggesting an autophagy-dependent cell death. Pretreatment with ICA increased cell viability by upregulating Blc-2 and prevented excessive autophagy by reducing Beclin-1 and LC3-II levels. A similar effect was observed when 3-methyladenine (3-MA), an inhibitor of PI3K and autophagosome formation, was administered in the same model [162], indicating that ICA attenuates ROS-driven autophagy death [141]. In vivo evidence aligns with these findings. In a neonatal C57BL/6 model of hypoxic–ischemic brain damage (4 h induction), decreased Beclin-1 and LC3-II expression alongside increased p62 levels were observed, consistent with impaired autophagy [27]. ICA pretreatment ameliorated hypoxic–ischemic brain damage pathology by preventing these changes and concurrently upregulating the estrogen receptors ERα and ERβ. Importantly, administration of 3-MA abolished ICA’s protective effects, suggesting that ICA modulates macroautophagy in this context [27]. Notably, ICA’s effects were absent when animals were treated with mitochondrial processing peptidase (MPP) or PHTPP, selective antagonists of ERα and ERβ, respectively, indicating that ICA’s actions are at least partly mediated through estrogen receptor signaling [27] (Table 4).

Cellular senescence is driven by multiple mechanisms and exhibits diverse phenotypes, with autophagy initially proposed as a suppressor of senescence [170]. Experimentally, administration of D-galactose is widely used to induce accelerated cellular senescence in vitro and in vivo [171]. In an acute PC12 cell model, exposure to 200 mM D-galactose for 48 h significantly reduced cell viability and increased senescence markers such as p21 and senescence-associated β-galactosidase. These changes were accompanied by marked changes in autophagy-related proteins, including an increased LC3II/I ratio, decreased p62 levels, and the upregulation of Atg7, Atg5, and Beclin-1 proteins, suggesting an abnormal activation of autophagy flux. This dysregulation was likely a consequence of mitochondrial dysfunction, mitochondrial permeability transition pore (mPTP) opening, and ROS accumulation. Treatment with ICA effectively mitigated these effects by reducing ROS generation, autophagy marker expression, and cell death. Notably, the protective action of ICA seemed to be dependent on its ability to attenuate mPTP permeability and consequently promote autophagic homeostasis [172]. In vivo studies using the SAMP8 and ceramide-based senescence mice model further support ICA’s anti-senescent effects. Chronic ICA administration improved memory performance and reduced senescence markers such as SA-β-Gal and p21 in a p53-mediated process [173]. Moreover, ICA treatment reduced p62 and LC3-II protein expression, decreasing autophagosome formation and suggesting that ICA prevents senescence not by enhancing, but by modulating autophagic flux [35]. Collectively, these findings suggest that ICA may exert neuroprotective effects by fine-tuning autophagy to prevent maladaptive overactivation rather than by broadly increasing autophagic activity. More detailed information from studies related to this topic is found in Table 4.

## 6. Excitotoxicity and ICA

Excitotoxicity arises from an imbalance in excitatory–inhibitory neurotransmission, most commonly due to excessive glutamate, which leads to overstimulation of the postsynaptic neurons. This process triggers an abnormal influx of calcium ions and the activation of pro-apoptotic pathways involving p53, JNK/p38, and caspase-3 [107]. Exposure to high glutamate concentrations results in neuronal injury, a phenomenon strongly implicated in the pathogenesis of neurodegenerative diseases [174]. Recent evidence identifies ICA as a potential neuroprotective agent against excitotoxic cell death. In human SH-SY5Y neuroblastoma cells exposed to 10 mM glutamate, ICA treatment conferred dose-dependent protection by restoring antioxidant defenses; limiting intracellular calcium accumulation; upregulating phosphorylated ERK, CREB, and CaMKIIα; and reducing apoptosis-related protein expression [26]. Beyond glutamate-induced toxicity, ICA also demonstrated neuroprotection against other excitotoxic insults. In a methylmercury-induced model of amyotrophic lateral sclerosis, a motor neuron degenerative disease, ICA enhanced the expression of SIRT-1, Nrf-2, and HO-1 while decreasing TNF-α and IL-1β levels [85]. Similarly, in ibotenic acid-induced excitotoxicity, ICA increased the Bcl-2/Bax ratio and suppressed phosphorylation of Erk 1/2, JNK, and p38 proteins, all members of the MAPK family [163]. In another study using albino Wistar rats, ICA ameliorated ammonia–glutamate-induced excitotoxicity via its antioxidant properties and stimulation of the NO/cGMP pathway [164]. Collectively, these studies show how ICA can be a powerful agent against excitotoxicity and its death pathways.

## 7. ICA Regulates Endoplasmic Reticulum Stress Under Pathological Conditions

The endoplasmic reticulum is a dense, membrane-bound organelle that plays a pivotal role in calcium storage and in the synthesis of lipids and proteins, accounting for the production of more than one-third of all cellular proteins [175]. Within its lumen, newly synthesized proteins undergo proper folding and, frequently, post-translational modifications before being transported to their final destinations. Under pathological conditions such as oxidative stress, nutrient deprivation, or inflammation, the protein-folding capacity of the endoplasmic reticulum (ER) can become overwhelmed, leading to the accumulation of misfolded or unfolded proteins. This accumulation triggers a condition known as ER stress, which, in turn, activates the unfolded protein response (UPR), a signaling network able to restore ER homeostasis. The unfolded protein response enhances the ER’s folding capacity and reduces the burden of new protein synthesis through three primary sensors: PERK (protein kinase RNA-like endoplasmic reticulum kinase), IRE1α (inositol-requiring enzyme 1 alpha), and ATF6 (activating transcription factor 6) [176]. Under stress conditions, the dissociation of chaperones such as binding immunoglobulin protein (BiP)/GRP78 from these sensors enables their activation. IRE1α and PERK undergo trans-autophosphorylation, initiating signaling cascades that activate transcription factors including XBP1 and ATF4, respectively. PERK further attenuates global translation via phosphorylation of the initiation factor eIF2α, thereby reducing the load of emerging polypeptides entering the ER. In addition, IRE1α mediates regulated IRE1-dependent decay (RIDD), which degrades mRNA in order to reduce ER stress levels. ATF6, in turn, is transported to the Golgi apparatus, where it is cleaved by site-1 and site-2 proteases (S1P and S2P), releasing a soluble cytosolic fragment that functions as a transcription factor [177]. ER stress can also be counterbalanced by endoplasmic reticulum-associated degradation (ERAD) and reticulophagy. In the former case, misfolded or unfolded proteins are transported from the endoplasmic reticulum lumen to the cytosol through membrane-associated transporters, where they are ubiquitinated and subsequently degraded by proteasomes. In reticulophagy, segments of the endoplasmic reticulum containing unfolded or misfolded proteins are delivered to and fused with lysosomes, where they are subsequently degraded by acidic hydrolases [176,178]. However, if ER stress persists or becomes excessive, adaptive responses give way to apoptotic signaling. For example, these sensors share the ability to increase CHOP (C/EBP-homologous protein) expression. CHOP is a transcription factor that regulates the expression of pro-apoptotic proteins such as BAX, Bcl-2-interacting mediator of cell death (BIM), and PUMA, while suppressing anti-apoptotic proteins, including BCL-2 and BCL-XL, thereby promoting activation of the intrinsic apoptotic pathway [176,179]. Prolonged activation of IRE1α converts it into a signaling scaffold through its association with TNF-associated factor 2 (TRAF2) and ASK1, leading to JNK and p38 activation and the induction of mitochondria-dependent cell death via modulation of pro-apoptotic proteins such as BAX, BAK, BIM, and BH3-interacting domain death agonist (BID) [179]. The recruitment of TRAF2 by IRE1α also results in the dissociation of TRAF2 from caspase-12, which is localized at the ER membrane. Once released from TRAF2, caspase-12 undergoes facilitated activation, triggering the activation of caspase-9 and caspase-3 in a cytochrome c- and apoptotic protease activating factor 1 (Apaf-1)-independent manner. In addition, caspase-12 can be cleaved by calpains activated by calcium release from the endoplasmic reticulum lumen during ER stress [179]. Although prolonged ER stress is a classical inducer of apoptotic cell death, a growing body of evidence indicates that ER-induced cell death may also proceed through ferroptosis (see the Section 8). In this context, an imbalance in calcium and iron homeostasis induced by ER stress culminates in the production of ROS and the execution of ferroptosis [178].

Thus, pharmacological or molecular attenuation of ER stress represents a promising therapeutic strategy across a broad spectrum of diseases. Although relatively underexplored, current evidence indicates that the neuroprotective effects of ICA against neuronal loss are mediated, at least in part, through the inhibition of ER stress. ICA has been reported to attenuate ER stress in several experimental contexts, including models of Alzheimer’s disease [130], MCAO [80], SCI [33], OGD/R [138], and pharmacologically-induced ER stress [131,165]. Across these models, ICA treatment led to a marked reduction in the expression of key ER stress markers and associated apoptotic mediators, including GRP78, phosphorylated PERK (p-PERK), IRE1α, phosphorylated eIF2α (p-eIF2α), ATF4, XBP1, CHOP, and cleaved caspase-12 [130,131,165].

In rat hippocampal and cortical neuron cultures in which ER stress was triggered by corticotropin-releasing hormone (CRH) or OGD/R, ICA treatment effectively prevented ER stress progression by inhibiting NF-κB activation and, consequently, reducing the associated inflammatory response [138,166]. In PC12 cells, ICA administration for 24 h activated the transcription factor NFE2L1, leading to increased expression of synoviolin, an ER-anchored E3 ubiquitin ligase responsible for degrading misfolded proteins [131]. This ICA-induced upregulation of synoviolin conferred significant protection against ER stress-mediated apoptosis, an effect that was abolished when synoviolin expression was silenced via siRNA [131].

**Figure 5:**
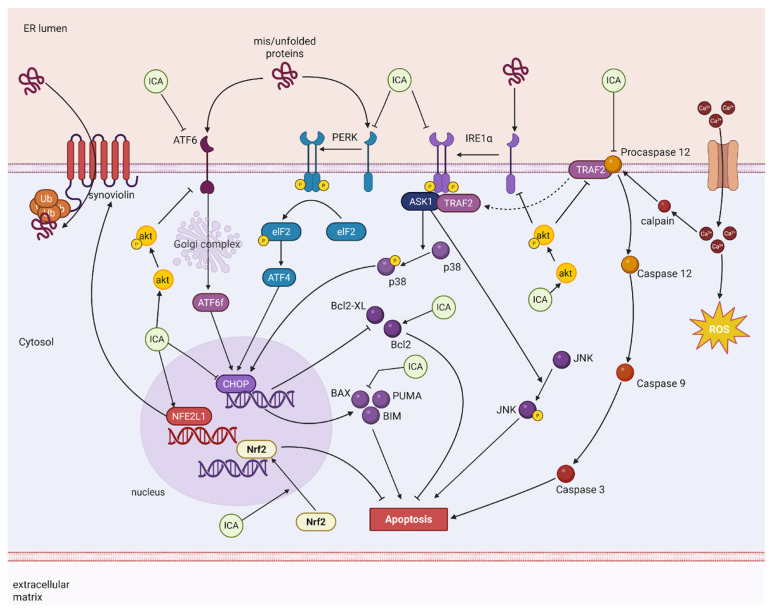
ICA acts on multiple molecular targets to prevent apoptosis induced by ERS (endoplasmic reticulum stress). Misfolded or unfolded proteins in the ER are retrotranslocated to the cytosol and ubiquitinated (Ub) by synoviolin. ICA stimulates NFE2L, which may further promote synoviolin expression. Excess accumulation of misfolded proteins in the ER activates the unfolded protein response (UPR) sensors ATF6, PERK, and IRE1α. ATF6 translocates to the Golgi apparatus, where it is cleaved into its active form (ATF6f), whereas PERK and IRE1α are activated through autophosphorylation. Activation of both sensors leads to increased CHOP (C/EBP-homologous protein). CHOP promotes the transcription of pro-apoptotic factors, such as BAX, BIM, and PUMA, while suppressing anti-apoptotic proteins, including Bcl2 and Bcl-XL. ICA inhibits IRE1α and ATF6 activation by activating the pAkt pathway, PERK activation, and CHOP expression, while increasing Bcl2 and reducing BAX expression. IRE1α can also form a signaling platform with ASK1 and TRAF2. ASK1 activates p38 MAPK and JNK, promoting CHOP expression and apoptosis. The dissociation of TRAF2 from procaspase-12 located in the ER membrane favors its activation, thereby promoting the proteolytic cleavage of caspases. ICA can inhibit caspase-12 activation and block apoptosis through this pathway. ER calcium release into the cytosol increases ROS production and promotes calpain activation. Calpain promotes the conversion of procaspase-12 into active caspase-12, which subsequently activates caspase-9 and caspase-3, leading to apoptosis. Furthermore, ICA promotes the nuclear translocation of Nrf2, enhancing antioxidant and anti-apoptotic responses.

Another molecular target implicated in ICA’s regulation of ER stress is the PI3K/Akt signaling pathway. In a mouse model of SCI, ICA administration (50 µmol/kg/day) prevented injury-induced upregulation of IRE1α, ATF6, XBP1, eIF2α, GRP78, and CHOP. The ER stress induced by thapsigargin in cultured spinal cord neurons was also blocked by pretreatment with ICA in a PI3K/AKT pathway-dependent manner. The PI3K inhibitor LY294002 abolished ICA’s protective effect, indicating that the suppression of ER stress and the promotion of cell survival by ICA are dependent on PI3K/Akt signaling [33]. In addition to modulating intracellular signaling, ICA may directly interact with ER stress-related proteins. Molecular docking analyses demonstrated ICA’s ability to bind to the active sites of GRP78, IRE1α, and PERK, with calculated binding free energies of −8.87, −9.80, and −10.35 kcal/mol, respectively. ICA formed hydrogen bonds with Asp34 and Thr37 of GRP78; Glu651 and Cys645 of IRE1α; and Asp955, Cys891, and Met934 of PERK. These interactions suggest that ICA can directly regulate these key ER stress sensors, thereby suppressing ER stress-induced injury in models of ischemic stroke [80] (Figure 5). For more detailed information about studies discussed in the present topic, see Table 4.

Together, these findings indicate that ICA may exert its beneficial effects in CNS cells by preventing ER stress triggered by different insults, thereby advancing understanding of this flavonoid’s mechanism of action. However, further studies are needed to better elucidate the molecular mechanisms underlying ER stress inhibition.

## 8. Role of ICA in Ferroptosis

Ferroptosis is a non-apoptotic, regulated form of cell death that plays a pivotal role in degenerative diseases and malignancies [180]. It is an iron-dependent process characterized by extensive peroxidation of polyunsaturated fatty acids (PUFAs) within membrane phospholipids, leading to membrane destabilization and rupture independently of caspase activation [180,181]. The iron metabolism plays an important role in the execution of ferroptosis. Extracellular iron bound to transferrin is internalized via endocytosis through its interaction with Transferrin Receptor 1 (TfR1). Within endosomes, ferric iron (Fe^3+^) is reduced to ferrous iron (Fe^2+^) and exported to the cytosol by metal transport proteins (such as divalent metal transporter 1 (DMT1)) [180,182,183]. Subsequently, Fe^2+^ is sequestered within ferritin for storage, a process facilitated by interactions with poly(rC)-binding proteins [180]. This cytosolic Fe^2+^ is central to ferroptotic mechanisms, as it fuels the Fenton reaction, generating ROS such as peroxyl and hydroperoxyl radicals. These radicals catalyze the peroxidation of PUFAs, triggering a self-propagating chain reaction across the plasma membrane. Additionally, this process can be initiated by non-enzymatic Fenton-type reactions or enzymatically via lipoxygenases [180,184].

Another hallmark of ferroptosis is the reduction in antioxidant enzymes, particularly glutathione peroxidase 4 (GPX4), an enzyme that converts phospholipid peroxides into lipid alcohols, with expression controlled by selenium and GSH [185]. GSH is an essential cofactor for peroxidases, inhibiting lipid peroxidation and limiting the Fenton reaction by reducing hydroxyl radicals. GSH biosynthesis is regulated by cysteine metabolism, which involves cysteine acquisition via system X_c_^−^ or de novo synthesis through the trans-sulfuration pathway [180,186]. Once inside the cell, cysteine is processed by glutamate–cysteine ligase (GCL) and GSH synthetase (GSS) to generate the γ-glutamyl-cysteinyl-glycine tripeptide. In this way, ferroptosis inducers like erastin and sulfasalazine inhibit system X_c_^−^, preventing cystine import, while RAS-selective lethal 3 (RSL3) and statins induce ferroptosis via GPX4 inhibition [187,188].

Overexpression of ferritin heavy chain 1 (FTH1) in PC12 cells exposed to 6-OHDA, classically used to reproduce in vivo models of Parkinson’s disease, blocks ferroptosis and improves cell viability [189]. In a ferroptosis model induced by RSL3 in murine hippocampal HT22 cells, several flavonoids conferred protection. In contrast, ICA failed to prevent ferroptotic loss of cell viability, although only a single dose of 10 μM was tested, and higher concentrations may be required to prevent cell death [167]. Nevertheless, emerging evidence suggests a potential link between ICA and Mouse Double Minute 2 (MDM2)/Mouse Double Minute X (MDMX), negative regulators of p53 that promote ferroptotic death by altering membrane lipid composition. Inhibition of the MDM2/MDMX complex has been shown to lead to an accumulation of monounsaturated lipids, reduced CoQ_10_, and modify acylcarnitines and tri/diacylglycerols [190]. Network pharmacology analyses have identified MDM2 as a putative ICA target [36]. In APP/PS1 Alzheimer’s disease mice (10 months old), both ICA treatment and MDM2 knockdown prevented memory impairment, reduced intracellular iron accumulation, and restored antioxidant capacity [36]. Moreover, ICA and its glycosides effectively inhibit lipid peroxidation [191]. Specifically, ICA alone mitigates iron overload-induced mitochondrial damage through the modulation of ERK1/2/JNK-MAPK and PI3K/AKT/mTOR signaling pathways in bone marrow stromal cells [142,192]. Although the available evidence remains sparse, these findings collectively suggest that ICA may prevent ferroptotic cell death in neurodegenerative contexts, potentially through mechanisms involving iron homeostasis, antioxidant restoration, and MDM2 modulation. Further investigation into how ICA modulates ferroptosis-related markers—specifically ferritin, TfR1, GPX4, and the system x_c_^−^—is required to elucidate its therapeutic mechanism, as existing studies suggest its active role in regulating these pathways [168,169].

## 9. Conclusions

Traditional knowledge represents an invaluable resource for the identification of bioactive molecules with therapeutic potential, many of which are now globally available as dietary supplements, reflecting the widespread adoption of these practices. However, confirmation of these promising effects with scientific studies is important to ensure adequate, secure, and efficient use in patients across different disease contexts. ICA, a polyphenolic compound present in Epimedium-based formulations, has demonstrated broad systemic protective effects in a wide range of experimental models. In this review, we synthesized the molecular mechanisms through which ICA modulates neural cell death pathways. ICA attenuates neuroinflammation by reducing the expression of inflammatory mediators, including TNF-α, IL-1β, IL-6, iNOS, and COX-2, and by suppressing key signaling cascades such as NF-kB and TLR4. Interestingly, ICA also seemed to modulate the immune system systemically, by inhibiting Th1 and Th17 cell differentiation and suppressing inflammatory infiltration in the CNS. Therefore, ICA’s anti-inflammatory impact appears to be robust, modulating local and systemic responses. In this context, more studies are necessary to better describe and to confirm the contribution of systemic or local inflammatory modulations to ICA’s neuroprotective benefits in neurodegenerative diseases. In parallel, ICA counteracts oxidative stress by consistently reducing ROS, NO, and lipid peroxidation, while promoting Nrf2 nuclear translocation and the upregulation of antioxidant enzymes such as HO-1 and NQO1. This antioxidant response exhibits extensive crosstalk with inflammatory signaling and contributes to the modulation of excitotoxicity, ferroptosis, ER stress, and autophagy.

Preclinical evidence consistently indicates a robust neuroprotective effect of ICA against apoptosis in different models that mimic CNS pathologies. Its action is predominantly associated with the modulation of anti- and pro-apoptotic proteins, such as Bcl-2 and BAX, respectively. Additional evidence suggests that icariin inhibits apoptosis by reducing ER stress. However, other regulatory factors of the apoptotic pathway may also be modulated by ICA but have not yet been investigated. In this context, inhibitors of apoptosis proteins (IAPs), which suppress caspase activity, could potentially be upregulated by ICA. Another pro-apoptotic member of the Bcl-2 family that has gained increasing attention in recent years is Bcl-2-related ovarian killer (BOK). BOK is localized to the endoplasmic reticulum membrane and is capable of inducing ER stress-dependent apoptosis as well as mitochondrial outer membrane permeabilization independently of BAX and BAK. Therefore, evaluating the potential effects of ICA on BOK may provide novel and relevant insights into the mechanisms underlying the flavonoid’s modulation of cell death.

Another emerging topic in the literature is the inhibitory effect of ICA on ferroptosis-induced cell death. Evidence in the CNS remains limited and requires further investigation. There are currently no data demonstrating that ICA regulates components of system Xc^−^, a cystine transporter essential for glutathione synthesis, nor are there data regarding its effects on proteins involved in iron metabolism, such as ferritin and transferrin. Ferroptosis depends on polyunsaturated fatty acids in biological membranes, which are incorporated into these structures through specific enzymatic pathways. It is plausible that the inhibition of ferroptosis by ICA involves the regulation of these critical molecular targets required for the initiation of this form of cell death. Accordingly, investigating these pathways is essential to better understand the relationship between ICA and ferroptosis.

ICA further enhances cell survival by activating pro-survival signaling pathways, most notably PI3K/Akt/GSK-3β axis, with multiple studies indicating a strong dependence on SIRT-1 activation. As discussed here, computational modeling studies suggest that ICA can directly bind to proteins extremely important in neurodegenerative diseases, such as BDNF-related signals, RAGE, and glutamate ionotropic receptor AMPA type subunit 1. Therefore, besides the consistent antioxidant, anti-inflammatory, and anti-apoptotic signaling properties in the nervous system, ICA could promote neuroprotection by directly modulating key targets of neurodegeneration. However, the interpretation of these effects is complicated by the lack of standardization across experimental models, heterogeneous routes of administration, and wide variability in dosing regimens. Importantly, these limitations should be considered within a pharmacokinetic–pharmacodynamic framework. Low oral bioavailability, rapid systemic clearance, and limited neural tissue distribution impose constraints on ICA’s application as a conventional CNS-targeted therapeutic agent. The apparent requirement for higher doses in CNS-related models, compared with peripheral or ocular targets, likely reflects pharmacokinetic barriers rather than reduced intrinsic efficacy. In this context, extensive metabolism and the generation of bioactive derivatives, such as icariside I, icariside II, and icaritin, may substantially contribute to the neuroprotective effects attributed to ICA in vivo.

Despite the limitations of the preclinical evidence, the data are robust and support ICA as a promising candidate for neurodegenerative disorders. However, clinical research in neurodegenerative disease contexts remains largely unexplored. To date, only a single uncontrolled clinical study has reported a reduction in depressive symptoms in patients with bipolar disorder and comorbid alcohol use disorder following ICA administration [133]. Moreover, current clinical and translational studies rarely address the contribution of ICA metabolites or define outcome measures relevant to CNS disorders.

Collectively, current evidence indicates that ICA does not operate as a single-target neuroprotective agent but rather as a pleiotropic modulator whose efficacy is shaped by dose, tissue accessibility, metabolic conversion, and cellular context. Future investigations should therefore move beyond descriptive efficacy and prioritize direct comparisons between ICA and its major metabolites, dose-dependent and tissue-specific analyses, and pharmacokinetically informed experimental designs. Addressing these challenges will be essential to translate robust preclinical neuroprotection into clinically meaningful applications.

## Figures and Tables

**Table 1:** Effects of ICA on Inflammation in Neurodegenerative models.

Disease Model	Animal Model /Cell	Dose/ Concentration	Administration, Time of Exposure	Observed Effect	Ref.
AD	APP/PS1-21 transgenic mice (C57BL/6J)	100 mg/kg	Gavage; daily for 10 days	Reduced neuroinflammatory reaction; decreased TGF-β1 immunoreactivity in cortex/hippocampus and Iba-1 levels.	[77]
MS (EAE)	C57BL/6 mice	25 mg/kg	Oral; daily from day 5 to 15	Inhibited Th1 and Th17 cell differentiation; suppressed inflammatory infiltration in the CNS.	[72]
Ischemic Stroke	Sprague Dawley Rats (MCAO)	10 and 30 mg/kg	Gavage; twice/day for 3 days	Decreased pro-inflammatory cytokines IL-1β and TGF-β1 in the cortex; blocked NF-κB activation via up-regulation of PPARα/γ.	[90]
MS (EAE)	C57BL/6 (female)	50, 150, 300 mg/kg	Gavage; daily for 6 days	Modulated HPA function and up-regulated GR (Glucocorticoid Receptor) and ERβ; noted increase in IL-17 (serology) despite symptom amelioration.	[69]
Ischemia (OGD)	PC12 cells	10^−7^–10^−5^ mol/L	Co-treatment; 2 h	Reduced HIF-1α, HSP-60, and HSP-70 expression (inflammatory/stress markers).	[91]
SCI	C57BL mice	20 and 50 μmol/kg	Oral; daily for up to 42 days	Attenuated IL-1β, TNF-α, and iNOS levels at 24 h and 3 days post-injury in the spinal cord homogenate; reduced edema.	[84]
Neuroinflammation (LPS)	C57BL/6J mice	20 mg/kg	Gavage; daily for 4 weeks	Decreased protein and gene expression of HMGB1, TNF-α, IL-1β, and RAGE in the hippocampus and serum; suppressed HMGB1-RAGE.	[63]
PD	WT and Nrf2 KO Mice	60 mg/kg	Intragastric; daily for 10 days	Decreased TNF-α and iNOS in the substantia nigra and in glial culture; attenuated glia-mediated neuroinflammation via Nrf2 pathway.	[83]
Neuroinflammation (LPS)	BV2 Microglia	0.1 μM	Pre-treatment 30 min	Decreased NO, IL-1β, and IL-18 in glial culture; inhibited microglia-mediated inflammation via Nrf2 activation.	[82]
SCI	Sprague Dawley Rats	30 μmol/kg	Oral; 1 h post-SCI then daily (7 days)	Reduced biochemical inflammatory index cytokines; reduced ROS and MDA.	[64]
MS (Relapse-Remit)	SJL/J mice (PLP induced)	12.5 and 25 mg/kg	Gavage; daily for 26 days	Decreased TNF-α, IL-6, iNOS, TGF-β, and NF-κB in the brain and spinal cord; reduced microglia activation.	[70]
Neuronal Inflammation	Primary Astrocytes (LPS)	10 μM	Pre-treatment 30 min	Inhibited TNF-α, IL-1β, COX-2, and iNOS via IGF-1 receptor signaling in astrocyte primary culture.	[88]
Neuropathic Pain	Wistar Rats (PSNL)	50 and 100 mg/kg	Gavage; daily for 21 days	Decreased TNF-α and IL-6; inhibited NR2B and TRPV1 receptors in spinal cord.	[73]
Ischemic Stroke	Sprague Dawley Rats	60 mg/kg	Intragastric; daily for 28 days	Reduced IL-6, TNF-α, and NF-κB in the brain; increased PPARα/γ (enhanced hypothermia protection).	[34]
Uveitis (EAU)	C57BL/6J/HMC3 cells	10 mg/kg (vivo)/0.01–20 μM (vitro)	Intragastric (7 days)/24 h (vitro)	Shifted microglia from pro-inflammatory to resolutive phenotype; decreased TNF-α, COX-2, iNOS; increased IL-10, CD206 in HMC3cell line.	[71]
Ischemic Stroke	Sprague Dawley Rats	10, 20, 40 mg/kg	Gavage; daily for 4 days	Decreased IL-1β in the brain; reduced microglial activation and ER stress-mediated inflammation.	[80]
Epilepsy	Mice (Pilocarpine model)	20 mg/kg	i.p.; daily for 7 days	Promoted microglial polarization of microglia from pro-inflammatory to resolutive.	[79]
POCD	Aged Sprague Dawley Rats	60 mg/kg	Gavage; daily for 7 days	Reduced TNF-α, IL-1β, IL-6 in the brain (CA1, CA3 and DG); inhibited TLR4/NF-κB signaling.	[75]
Vascular Dementia	Sprague Dawley Rats	20 mg/kg (Oral)/0.6 mg/kg (Nano)	Daily for 28 days	Reduced IL-1β, IL-6, and TNF-α in the Hippocampus.	[81]
MS (Cuprizone)	C57BL/6 Mice/BV-2	50 mg/kg (vivo)/15 μg/mL (vitro)	i.p. (14 days)/2 h pre (vitro)	Shifted microglia from pro-inflammatory to resolutive; reduced TNF-α, IL-1β via TLR4/NF-κB; increased IL-10 in microglial culture.	[92]
Ischemic Stroke	N2a cells (OGD)	10 μM	During OGD	Reduced TNF-α, IL-6, and IL-1β in N2a cell line.	[93]
ALS	Wistar Rats (Methylmercury)	15 and 30 mg/kg	Gavage; daily for 21 days	Mitigated neuroinflammation; reduced TDP-43 accumulation.	[85]

Abbreviations: AD (Alzheimer’s Disease), MS (Multiple Sclerosis), EAE (Experimental Autoimmune Encephalomyelitis), SCI (Spinal Cord Injury), PD (Parkinson’s Disease), POCD (Postoperative Cognitive Dysfunction), ALS (Amyotrophic Lateral Sclerosis), MCAO (Middle Cerebral Artery Occlusion), OGD (Oxygen-Glucose Deprivation), EAU (Experimental Autoimmune Uveitis), LPS (Lipopolysaccharide), PSNL (Partial Sciatic Nerve Ligation), TGF-β1 (Transforming Growth Factor beta 1), Iba-1 (Ionized calcium-binding adapter molecule 1), Th1/Th17 (T helper cells 1 and 17), CNS (Central Nervous System), IL-1β/IL-6/IL-10/IL-17/IL-18 (Interleukins), NF-κB (Nuclear Factor kappa-light-chain-enhancer of activated B cells), PPARα/γ (Peroxisome Proliferator-Activated Receptors alpha and gamma), HPA (Hypothalamic-Pituitary-Adrenal), GR (Glucocorticoid Receptor), ERβ (Estrogen Receptor beta), HIF-1α (Hypoxia-Inducible Factor 1-alpha), HSP-60/70 (Heat Shock Proteins 60 and 70), TNF-α (Tumor Necrosis Factor alpha), iNOS (Inducible Nitric Oxide Synthase), HMGB1 (High Mobility Group Box 1), RAGE (Receptor for Advanced Glycation End-products), WT (Wild Type), KO (Knockout), Nrf2 (Nuclear factor erythroid 2-related factor 2), NO (Nitric Oxide), ROS (Reactive Oxygen Species), MDA (Malondialdehyde), PLP (Proteolipid Protein), COX-2 (Cyclooxygenase-2), IGF-1 (Insulin-like Growth Factor 1), NR2B (N-methyl-D-aspartate Receptor Subunit 2B), TRPV1 (Transient Receptor Potential Vanilloid 1), CD206 (Cluster of Differentiation 206), TLR4 (Toll-Like Receptor 4), TDP-43 (TAR DNA-binding protein 43, CA1 (Cornu Ammonis area 1), CA3 (Cornu Ammonis area 3) DG (Dentate gyrus), and i.p. (Intraperitoneal).

**Table 2:** Effects of ICA in Oxidative Stress in Neurodegenerative models.

Disease Model	Animal Model /Cell	Dose/ Concentration	Administration, Time of Exposure	Observed Effect	Ref.
H_2_O_2_ toxicity	PC12 cells; H_2_O_2_	0.1–10 µM	Culture medium; 30 min pre + 1 h exposure	Reduced GSH depletion and DNA oxidation; inhibited JNK/p38 MAPK.	[107]
AD	SH-SY5Y neuroblastoma; Formaldehyde	1–10 µmol/L	Direct application; 4 h	Reduced Tau phosphorylation via GSK-3β inhibition.	[114]
AD	SAMP8 Mice	75 or 150 mg/kg	Orally; 15 days before collection	Stimulated SOD/GSH-Px; decreased MDA and NO.	[111]
AD	APP/PS1 Mice	120 mg/kg	Intragastric; 3 months	Reduced brain iron levels and pro-inflammatory cytokines.	[38]
SCI	Sprague Dawley Rats; induced injury	30 µmol/kg	Oral; 1 h post-SCI, daily for 7 days	Reduced ROS/MDA; augmented SOD/GSH; improved locomotion.	[64]
Diabetic Encephalopathy	PC12 cells; Diabetic Serum	5/10/20 µM	Culture medium; 48 h	Bound to Bax to inhibit mitochondrial migration; reduced ROS.	[109]
Excitotoxicity	SH-SY5Y cells; Glu	10^−6^–10^−4^ M	Culture medium; 24 h	Inhibited P47phox, iNOS, and p-JNK; anti-apoptotic.	[26]
ALS	Wistar Rats; MeHg+	15–30 mg/kg	Orally; Days 21–42	Modulated SIRT-1, Nrf-2, and HO-1; reduced TDP-43.	[85]
PD	PC12 cells; 6-OHDA	0.005–0.05 µM	Culture medium; 24 h pre + 24 h exposure	Activated Nrf2 signaling; decreased Bax/Bcl-2 ratio.	[106]
H_2_O_2_ toxicity	Primary neurons (mouse); H_2_O_2_	0.6–9.6 µM	Culture medium; Co-treatment; 1 h	Upregulated SIRT1-dependent CAT and Prx1.	[108]
PD (Rotenone)	PC12 cells/SD Rats	2–4 µM (Cell)/15–30 mg/kg (Animal)	Oral/Pre; 5 weeks (Animal)	Upregulated SIRT3/PGC-1α; restored DA neurons loss.	[82]
PD	WT and Nrf2 KO Mice; 6-OHDA	60 mg/kg (Animal)/0.1 µM (Cell)	Oral (10 days)/Culture (7 days)	Activated Nrf2 signaling; effects lost in Nrf2 KO.	[83]
MS	C57BL/6 Mice; CPZ Model	50 mg/kg (Animal)/15 µg/mL (Cell)	i.p. (14 days)/2 h pre (Cell)	Scavenged ROS; promoted remyelination via Nrf2/HO-1.	[92]
Vascular Dementia	In silico model	N/A	Molecular Docking/Dynamics	Targeted RAGE; effective docking with JUN, MAPK14, and IL-6.	[108]
AD	PC12 cells; NaN_3_ toxicity	0.01–1 µM	Culture medium; 2 h pretreatment + 24 h NaN_3_	Activated PI3K/Akt/GSK-3β; preserved MMP.	[112]
AD	Primary neurons	40–320 µg/mL	Culture medium; 24 h post-exposure	Upregulated CART via MAPK/ERK pathway.	[116]
AD	In silico model; Aβ42 toxicity	N/A	CDOCKER Molecular Docking	Capacity to bind BDNF and GRIA1.	[75]
Ischemia (Stroke)	Sprague Dawley Rats	10–30 mg/kg	Gavage; twice daily for 3 days	Increased PPARα/γ; inhibited NF-κB.	[90]
Ischemia	Primary neurons (rat); I/R	5–15 µM	8–24 h after reperfusion	Lowered ROS; maintained cellular Ca^2+^ homeostasis.	[101]
Ischemia	Sprague Dawley Rats; induced Stroke	5 mg/kg	Orally; 7 days pre + 14 days post	Reduced CA1 hippocampal apoptosis.	[104]
Epilepsy	Long-Evans Rats	75 mg/kg	i.p.; 1 h before insult	Modulated GluR2/ERK; reduced MDA; increased SOD.	[102]
SCI	C57BL Mice; induced injury	20–50 µmol/kg	Oral; daily (up to 42 days)	Restored GSH/SOD; downregulated Bax/Caspase-3.	[84]
Aging	C57BL/6J Mice	100 mg/kg	Gavage; 15 consecutive days	Activated Akt/Nrf2 and Sirtuins; restored youth-like GM.	[121]
PD	BV2 microglia; 6-OHDA	0.1 μM	Culture medium; 30 min pretreatment	Reduced oxidative stress markers, including nitric oxide (NO); enhanced nuclear translocation of Nrf2, leading to increased expression of antioxidant enzymes, HO-1 and NQO1; Nrf2 silencing reversed ICA effects.	[82]
PD	Primary neuron cultures; 6-OHDA	0.1 μM	Culture medium; 30 min pretreatment	Enhanced nuclear translocation of Nrf2, leading to increased expression of antioxidant enzymes HO-1 and NQO1.	[82]

Abbreviations: H_2_O_2_ (Hydrogen Peroxide), AD (Alzheimer’s Disease), SAMP8 (Senescence-Accelerated Mouse Prone 8), APP/PS1 (Amyloid Precursor Protein/Presenilin 1), SCI (Spinal Cord Injury), Glu (Glutamate), ALS (Amyotrophic Lateral Sclerosis), MeHg+ (Methylmercury), PD (Parkinson’s Disease), 6-OHDA (6-Hydroxydopamine), WT (Wild Type), KO (Knockout), MS (Multiple Sclerosis), CPZ (Cuprizone), i.p. (Intraperitoneal), NaN_3_ (Sodium Azide), Aβ42 (Amyloid-beta 42), I/R (Ischemia/Reperfusion), GSH (Glutathione), JNK (c-Jun N-terminal Kinase), MAPK (Mitogen-Activated Protein Kinase), GSK-3β (Glycogen Synthase Kinase-3 Beta), SOD (Superoxide Dismutase), GSH-Px (Glutathione Peroxidase), MDA (Malondialdehyde), NO (Nitric Oxide), ROS (Reactive Oxygen Species), Bax (Bcl-2-associated X protein), iNOS (Inducible Nitric Oxide Synthase), SIRT-1 (Sirtuin 1), Nrf-2 (Nuclear Factor Erythroid 2-related Factor 2), HO-1 (Heme Oxygenase-1), TDP-43 (TAR DNA-binding protein 43), Bcl-2 (B-cell lymphoma 2), CAT (Catalase), Prx1 (Peroxiredoxin 1), SIRT3 (Sirtuin 3), PGC-1α (Peroxisome Proliferator-activated Receptor Gamma Coactivator 1-alpha), DA (Dopaminergic), RAGE (Receptor for Advanced Glycation End-products), JUN (Jun Proto-Oncogene), MAPK14 (Mitogen-Activated Protein Kinase 14), IL-6 (Interleukin 6), PI3K (Phosphoinositide 3-kinase), Akt (Protein Kinase B), MMP (Mitochondrial Membrane Potential), CART (Cocaine- and Amphetamine-Regulated Transcript), ERK (Extracellular Signal-Regulated Kinase), BDNF (Brain-Derived Neurotrophic Factor), GRIA1 (Glutamate Ionotropic Receptor AMPA Type Subunit 1), PPARα/γ (Peroxisome Proliferator-Activated Receptor Alpha/Gamma), NF-κB (Nuclear Factor Kappa-light-chain-enhancer of Activated B cells), Ca^2+^ (Calcium Ion), GluR2 (Glutamate Receptor 2), GM (Gut Microbiota), and NQO1 (NAD(P)H Quinone Dehydrogenase 1).

**Table 3:** Effects of ICA on Apoptosis in Neurodegenerative models.

Disease Model	Animal Model /Cell	Dose/ Concentration	Administration, Time of Exposure	Observed Effect	Ref.
AD	PC 12 cells induced by Aβ25–35	5–10 µM	30 min pretreatment/6 h treatment	Decreased Aβ25–35-induced cytotoxicity and apoptosis rate; inhibiting tau protein hyperphosphorylation at Ser396, Ser404 and Thr205 sites	[128]
AD	PC 12 cells induced by Aβ25–35	2.5–20 µM	1 h pretreatment	Decreased Aβ25–35-induced apoptosis	[129]
AD	PC 12 cells induced by sodium azide (NaN3)	0.01–1 µM	2 h pretreatment	Reduced NaN3-induced cell damage; reduced the leakage rate of LDH; increased the MMP; decrease in glucose concentration, indicating increased glucose consumption; Tau phosphorylation at the Ser396/404 and Thr217 sites significantly decreased	[112]
AD	APP/PS1 mice	60 mg/kg	Orally administered with ICA daily for 3 months	Reduced neuronal apoptosis by suppressing the ER stress signaling pathway; improved behavioral performance	[130]
AD	APP/PS1/Tau triple-transgenic mice	60 mg/kg	Intragastrically administered daily for 5 months	Memory deficits improved; neuronal and synaptic damage mitigated; reduced amyloid-β accumulation and tau hyperphosphorylation; deficits of proteins in the insulin signaling pathway and their phosphorylation levels were significantly reversed	[29]
AD	Tg2576 mice	60 mg/kg	Orally administered daily for 3 months	Decreased the levels of Aβ and APP; enhanced neurogenesis; improved memory function	[131]
AD	SAMP8 mice	60 mg/kg	Intragastric administration for 22 days	Reverse learning and memory impairment; reduce the expression of cytotoxic Aβ1-42; increased the expression of anti-apoptotic protein Bcl-2; decreased the expression of pro-apoptotic protein Bax	[67]
AD	SH-SY5Y (Formaldehyde)	1–10 µM	4 h exposure	Attenuated apoptotic nuclear disintegration and cell death	[114]
PD	PC12 cells induced by 6-OHDA and Sprague Dawley rats	0.1 µM/20 mg/kg	24 h pretreatment/injection in the brain	ameliorated the development of LID/lowered AIM scores attenuated neuroinflammation	[25]
PD	Wistar rats induced by Haloperidol	100 mg/kg	Oral administration 30 min after the administration of Haloperidol daily	Decreasing the neurotoxicity via lowering lipid peroxidation NO, GSK-3β Contents; increasing antioxidant biomarkers, TH, and recovering monoamines contents	[132]
PD	C57BL/6 mice induced by MPTP	50–200 mg/kg	3 days pretreatment by intragastric administration	Ameliorate the decreased striatum DA content; decreased loss of TH-IR neurons; reversed changes of Bcl-2, Bax and caspase 3 protein expressions	[133]
PD	PC12 cells (6-OHDA induced)	0.005–0.05 µM	24 h pre + 24 h exposure	Inhibited apoptosis via marked decreases in Bax/Bcl-2 ratio, cytochrome c release, and Caspase-3 cleavage	[106]
DE	PC12 cells induced by AGEs	10–20 µM	48 h cotreatment	Inhibit Bax dimer formation and migration to mitochondria; inhibit apoptosis and oxidative stress	[109]
Corticosterone-induced cytotoxicity	Primary hippocampal neuronal cultures from Sprague Dawley rats	1–10 µM	2 h pretreatment	Reduced TUNEL-positive cell numbers; improved mitochondrial membrane potential; inhibition of caspase-3 activation	[134]
Depression	Sprague Dawley Rats less than 24 h old, Corticosterone-induced cell damage	0.1 and 1 µM	orally 2 h pretreatment with 1 µM corticosterone	Lowered Caspase 3 activation, ROS production, increased expression of p-AKT, and SOD activity	[135]
Cavernous nerve crush injury	12-week-old Sprague Dawley Rats, submitted to nerve crush injury.	1, 5 and 10 mg/kg	orally daily supplement or single dose 2 h prior to sacrifice.	Decreased nerve fiber loss, nNOS production, calponin and lowered expression of eNOS.	[136]
Neuropathic Pain	Wistar Rats	10, 50, 100 mg/kg	Orally daily for 21 days after surgery	Decreased expression of TNF-α, IL-6, Bax and increased expression of Bcl-2	[73]
Epilepsy	Long-Evans Rats	75 mg/kg	Injection 1 h before induction of hypoxia	Lowered stages of seizure by ameliorating changes in MDA, SOD, Bax/bcl-2 ratio, and Caspase-3. Ameliorating ERK II and GluR2 expression	[102]
Schizophrenia	Sprague Dawley Rats	50 mg/kg	Orally 3 times per day for 2 weeks	Regulates the effects of mTOR, ATP1B2 and miR-144-3p pathway	[137]
Hypoxic-ischemic brain damage	C57BL/6 neonatal mice	10 mg/kg	Intraperitoneally 20 min before hypoxia-ischemia	Promoted autophagy in hypoxic–ischemic brain-damaged mice, increasing Beclin1 and IC3-II, reducing p62 activation	[27]
Ischemic Stroke	Mouse neuroblastoma N2a cells	1, 5, 10, 20, 40, and 80 µM	15 min, during oxygen-glucose deprivation	Ameliorates the Bax/Bcl2 ratio, reduced caspase-3, TNF-α, IL-1β, IL-6 and PKM2	[93]
Ischemic Stroke	Sprague Dawley microglia and Primary cortical neurons	0.25, 0.5 and 1 mg/mL	1 h of pretreatment and 2 h of treatment	Inhibited apoptosis induced by OGD/R through IRE1/XBP1 signaling pathway	[138]
Neonatal hypoxic–ischemic brain damage	C57Bl/6J P7 mouse pups using Rice Vannucci method	10 mg/kg	intraperitoneally 20 min before ischemia	Reduced brain cerebral infarct volume and improved neurobehavioral recovery following hypoxic ischemic injury by reducing expression of cleaved caspase 3 and increasing p-Akt	[139]
Middle cerebral artery occlusion (MCAO	Cerebral Ischemia–Reperfusion (I/R) Injury in ICR mice	60 mg/kg	Intraperitoneally injection and 1 h of pretreatment	Ameliorated body weight loss, neurological injury, infarct volume, and pathological change in acute ischemic stroke mice, and protected against neuronal cell apoptotic death, oxidative and nitrosative stress, lipid peroxidation, and extracellular matrix (ECM) accumulation in the brains	[140]
MCAO	Ischemia-Reperfusion in middle cerebral artery and OGD-induced MCAO in primary cortical neuron culture	In vivo: 50, 100 and 200 mg/kg In vitro: 80 µM	In vivo: After day 1, 3, 7 of MCAO In vitro: After OGD treatment	Reduced infarct size, upregulating SIR1 and PCG-1α expression	[31]
MCAO	Sprague Dawley Rats with ischemia–reperfusion in middle cerebral artery	60 mg/kg	Daily for 28 days after MCAO surgery	Increased therapeutic hypothermia effect time in 20min, enhancing the inhibitory effect of mild hypothermia, lowering the expression of TNF-α, IL-6, p-JAK2, p-STAT3, C-caspase 3, regulating Bax/Bcl ratio and increasing PPARα, PPARγ, Nf-κβ and nuclear Nrf2	[34]
Hypoxic ischemic brain damage	neonatal SD rats, with hypoxia induced by hypoxia chamber	100 and 200 mg/kg	Daily for 7 days before hypoxia-ischemia One dose post HI induction	Lowered the expression of NF-κB, p65, IL-1β, TNFα, caspase-3 and 9	[66]
OGD/R	PC12 cells	10 µM	1 h before hypoxia	Increased cell viability, regulating Bax/Bcl-2 ratio, and lowering C-caspase 3, GAPDH and LC3-I and II expression	[141]
OGD/R	PC12 cells	0.1, 1 and 10 µM/L	1 h before hypoxia	Lowered HIF-1α, neuron-specific enolase, HSP-60 and HSP-70, increasing cell viability	[91]
Excitotoxicity	SH-SY5Y cells (Glutamate induced)	1–100 µM	24 h exposure	Anti-Apoptotic Signaling: Attenuated glutamate-induced apoptosis by inhibiting the JNK/p38 MAPK pathway; reduced Bax/Bcl-2 ratio and Cleaved Caspase-3/9	[26]
Ischemia (I/R)	Primary Cortical Neurons	5–15 µM	8–24 h post-reperfusion	Prevented apoptosis driven by intracellular Ca^2+^ overload; reduced ROS-dependent apoptotic signaling	[101]
SCI	C57BL Mice (Traumatic)	20, 50 µmol/kg	Oral; daily (42 days)	Downregulated ER stress apoptotic markers (CHOP, Caspase-12) and mitochondrial markers (Bax, Cyt C); reduced TUNEL+ neurons	[84]
Iron overload apoptosis	Bone marrow mesenchymal cell	10, 50 and 100 µmol	24 and 48 h	Reduced cleaved caspase-3, and apoptosis-related proteins while regulating Bax/Bcl-2 ratio; Regulates mitochondrial stability, reducing cytosolic cytochrome c	[142]

Abbreviations: AD (Alzheimer’s Disease), PD (Parkinson’s Disease), DE (Diabetic Encephalopathy), MDD (Major Depressive Disorder), SCI (Spinal Cord Injury), OGD/R (Oxygen-Glucose Deprivation/Reoxygenation), MCAO (Middle Cerebral Artery Occlusion), I/R (Ischemia/Reperfusion), PSNL (Partial Sciatic Nerve Ligation), HI (Hypoxia-Ischemia), APP/PS1 (Amyloid Precursor Protein/Presenilin 1), SAMP8 (Senescence-Accelerated Mouse Prone 8), 6-OHDA (6-Hydroxydopamine), MPTP (1-methyl-4-phenyl-1,2,3,6-tetrahydropyridine), NaN_3_ (Sodium Azide), Aβ (Amyloid-beta), AGEs (Advanced Glycation End-products), ER (Endoplasmic Reticulum), MMP (Mitochondrial Membrane Potential), LDH (Lactate Dehydrogenase), ROS (Reactive Oxygen Species), MDA (Malondialdehyde), SOD (Superoxide Dismutase), NO (Nitric Oxide), TH (Tyrosine Hydroxylase), DA (Dopamine), Bcl-2 (B-cell lymphoma 2), Bax (Bcl-2-associated X protein), Caspase (Cysteine-aspartic protease), Akt (Protein Kinase B), ERK (Extracellular Signal-Regulated Kinase), MAPK (Mitogen-Activated Protein Kinase), JNK (c-Jun N-terminal Kinase), GSK-3β (Glycogen Synthase Kinase-3 beta), PPAR (Peroxisome Proliferator-Activated Receptor), NF-κB (Nuclear Factor kappa-light-chain-enhancer of activated B cells), Nrf2 (Nuclear factor erythroid 2-related factor 2), TNF-α (Tumor Necrosis Factor alpha), IL-6 (Interleukin 6), IL-1β (Interleukin 1 beta), nNOS/eNOS (Neuronal/Endothelial Nitric Oxide Synthase), SIRT1 (Sirtuin 1), PGC-1α (Peroxisome proliferator-activated receptor gamma coactivator 1-alpha), IRE1/XBP1 (Inositol-requiring enzyme 1/X-box binding protein 1), TUNEL (Terminal deoxynucleotidyl transferase dUTP nick end labeling), LID (Levodopa-Induced Dyskinesia), AIM (Abnormal Involuntary Movement), and i.p. (Intraperitoneal).

**Table 4:** Effects of ICA in Other Cell Death Mechanisms in Neurodegenerative Models.

Disease Model	Cell Death Mechanisms	Animal Model /Cell	Dose/ Concentration	Administration, Time of Exposure	Observed Effect	Ref.
ALS	Excitotoxicity by MeHg+	Wistar Rats	15 and 30 mg/kg	Orally, daily for 11 days	Mitigated behavioral, biochemical, neurochemical, and gross morphological changes characteristic of an ALS-like phenotype, inhibited SIRT-1, HO-1 and TDP-43; promoted Nrf-2 migration to the nucleus	[85]
Excitotoxicity by Ibotenic Acid	Excitotoxicity by ibotenic acid	Sprague Dawley rats	20 and 40 mg/kg	Orally, twice a day for 20 days	Inhibited the phosphorylation of Erk, JNK, p38; regulated the bcl-2/bax ratio, inhibiting apoptosis	[163]
Excitotoxicity by glutamate	Excitotoxicity by glutamate	SH-SY5Y cells	10^−6^, 10^−5^ 10^−4^ M	24 h after treatment with glutamate	Increased p46 and iNOS; lowered p-JINK, Bax/Bcl and p-P38/P38 ratio and active caspase 3 and 9	[26]
Ischemic stroke	Endoplasmic Reticulum Stress	Sprague Dawley	10, 20, 40 mg/kg	4 days/once a day/Not specified	Inhibited microglial activation; attenuated neuronal damage; suppressed inflammatory signaling pathways mediated by ERS. Decreased IL-1B, reticulum stress, IRE1-alpha, p-ERK.	[80]
Ischemic stroke	Endoplasmic Reticulum Stress	Microglia and cortical neurons isolated from neonatal Sprague Dawley rats	0.25 mg/L 0.5 mg/L 1 mg/L	1 h pretreatment + 2 h co-treatment during OGD	Inhibited the IRE1α-XBP1 pathway; reduced C-caspase-3 levels; inhibited apoptosis. Inhibited apoptosis by inhibiting IRE1α-XBP1, reducing C-caspase-3 levels	[138]
Spinal cord injury	Endoplasmic Reticulum Stress	Mice C57BL/6 and spinal cord cell culture	50 um/kg	Once per day	Reduced ER by upregulating PI3k/AKT. Reduced ATF6, IRE1, GRP78, XBP1 and elF	[33]
ER stress	Endoplasmic Reticulum Stress	APP/PS1 transgenic mice	60 mg/kg body weight	Orally (gavage), 3 months (chronic treatment in 9-month-old mice).	Improved spatial memory, recognition memory; favored non-amyloidogenic APP processing; reduced downstream pro-apoptotic signals (TRB3, GADD34, ERO1α mRNA); reduced apoptosis; lowered caspase activation; restored Bax/Bcl-2 balance; preserved neuronal density; reduced Aβ burden and downregulated APP/BACE1 (β-secretase) while upregulating ADAM10 (α-secretase); suppressed GRP78 and PERK/eIF2α pathway	[130]
AD	Ferroptosis	APP/PS1 mice	5 and 10 mg/mL	Intragastrically administered daily for 5 weeks	Improved the neurobehavioral, memory, motor abilities; ameliorated neural damage	[36]
Hepatic encephalopathy (HE)	ammonia-glutamate excitotoxicity	Wistar rats	100 mg/kg	Orally administered for 14 days	Recovered of hepatic enzyme activities; improved locomotor and memory functions; reduced malondialdehyde, calcium, nitric oxide contents; downregulated lactate dehydrogenase activities	[164]
ER stress	Endoplasmic reticulum stress	PC12 cells	0.1 μmol/L	24 h, 48 h, 72 h	Relieved ERS; reduced apoptosis; down-regulated CHOP and Grp78 expression	[165]
ER stress	Endoplasmic reticulum stress	Primary hippocampal neurons of Sprague Dawley	10, 20, and 50 nM	2 h Pretreatment	Promoted BiP and IFN- γ expression; suppressed IRE-1α, XBP-1, NF-κβ, Il-1β and IL-6 and TNF-α; increased cell viability	[166]
Ferroptosis	RSL3-induced ferroptosis	HT22 cells	10 μmol	24 h after incubation with RSL3	Failed in preventing death by ferroptosis	[167]
Esophageal cancer	Primary esophageal epithelial cell	EC109, TE1 and HET-1A	20 40 or 80 μmol	12, 24 or 36 h in incubation	Inhibited ESCCs growth by activating ERS signaling; increased ROS; activated caspase 3/9	[125]
ER stress	Tunicamycin induced ER stress	PC12 cells	2.5, 5 and 10 μmol	24 h	Increased synoviolin expression; reduced ER stress-induced cell death	[20]
OA	Chondrocyte ferroptosis	Male Sprague Dawley rats and Human SW1353 chondrocytes	40 μmol and 80 mg/kg/d	24 h for in vitro/28 days for in vivo	Countered the IL-1β-induced upregulation of MMPs and ADAMTS-5; restored collagen II and SOX9 expression; reduced intracellular ROS, lipid ROS, MDA; alleviated Era-induced ferroptosis; reduced articular cartilage damage in OA rats	[168]
AD	Ferroptosis	APP/PS1 mice	5 mg/kg and 10 mg/kg	Intragastrically administered daily	Improve the neurobehavioral, memory and motor abilities of AD mice; lower ferroptosis level; enhanced oxidative stress resistance	[36]
Rheumatoid arthritis	Ferroptosis	Lipopolysaccharide (LPS)-induced synoviocytes	2, 5, 10 µmol	24 h pretreatment	Attenuated iron content; Increased activity of GPX4, MDA, Nrf2m, SLC3a2l and SLC7A11	[169]

Abbreviations: ALS (Amyotrophic Lateral Sclerosis), MeHg+ (Methylmercury), SIRT-1 (Sirtuin 1), Nrf-2 (Nuclear Factor Erythroid 2-Related Factor 2), HO-1 (Heme Oxygenase-1), TDP-43 (TAR DNA-Binding Protein 43), MAPK (Mitogen-Activated Protein Kinase), iNOS (Inducible Nitric Oxide Synthase), JNK (c-Jun N-terminal Kinase), Bax (Bcl-2-associated X protein), Bcl-2 (B-cell lymphoma 2), OGD/R (Oxygen-Glucose Deprivation/Reoxygenation), ER (Endoplasmic Reticulum), IRE1α (Inositol-Requiring Enzyme 1 Alpha), XBP1 (X-box Binding Protein 1), IL-1β (Interleukin 1 Beta), IL-6 (Interleukin 6), TNF-α (Tumor Necrosis Factor Alpha), PI3K (Phosphoinositide 3-Kinase), Akt (Protein Kinase B), ATF6 (Activating Transcription Factor 6), GRP78 (Glucose-Regulated Protein 78), eIF2α (Eukaryotic Initiation Factor 2 Alpha), APP/PS1 (Amyloid Precursor Protein/Presenilin 1), BACE1 (Beta-site APP Cleaving Enzyme 1), ADAM10 (A Disintegrin and Metalloproteinase Domain-containing Protein 10), TRB3 (Tribbles Homolog 3), GADD34 (Growth Arrest and DNA Damage-inducible Protein 34), ERO1α (Endoplasmic Reticulum Oxidoreductase 1 Alpha), TUNEL (Terminal deoxynucleotidyl transferase dUTP nick end labeling), HE (Hepatic Encephalopathy), CHOP (C/EBP Homologous Protein), ROS (Reactive Oxygen Species), OA (osteoarthritis) and ESCC (Esophageal Squamous Cell Carcinoma).

## Data Availability

No new data were created or analyzed in this study. Data sharing is not applicable to this article.

## Supplementary Materials

The following supporting information can be downloaded at: https://www.mdpi.com/article/10.3390/ijms27052247/s1.

## Abbreviations

The following abbreviations are used in this manuscript:

ICA	Icariin
SIRT3	Sirtuin 3
PRDX3	Peroxiredoxin 3
ROS	Reactive Oxygen Species
RNS	Reactive Nitrogen Species
BBB	Blood–brain barrier
PD	Parkinson’s Disease
AD	Alzheimer’s Disease
ER	Endoplasmic Reticulum
CNS	Central Nervous System
Nrf2	Nuclear Factor Erythroid 2–Related Factor 2
SIRT1	Sirtuin 1
Prx1	Peroxiredoxin 1
ARE	Antioxidant Response Element
ERα/ERβ	Estrogen Receptor Alpha/Beta
PERK	Protein Kinase RNA-like ER Kinase
GRP-78	Glucose-Regulated Protein 78
IRE1α	Inositol-Requiring Enzyme 1 Alpha
XBP1	X-Box Binding Protein 1
Bcl-2	B-cell Lymphoma 2
Bax	Bcl-2-Associated X Protein
PGC-1α	PGC-1 Alpha
C57BL/6	C57 Black 6
SJL/J	Swiss Jim Lambert/Jackson
TNF-α	Tumor Necrosis Factor Alpha
iNOS	Inducible Nitric Oxide Synthase
ERK1/2	Extracellular Signal-Regulated Kinases
p38	p38 MAPK
IL-6/IL-1β	Interleukin 6/1 Beta
HIF-1α	Hypoxia-Inducible Factor 1 Alpha
NMDA	N-Methyl-D-Aspartate Receptor
TRPV1	Transient Receptor Potential Vanilloid 1
GLUN2B	NMDA Receptor Subunit 2B
PNI	Peripheral Nerve Injury
NF-κB	Nuclear Factor Kappa B
p65	RelA Subunit of NF-κB
COX-2	Cyclooxygenase 2
p-AKT	Phosphorylated Protein Kinase B
IGF-1R	Insulin-Like Growth Factor 1 Receptor
LPS	Lipopolysaccharide
TLR4	Toll-Like Receptor 4
APP/PS1	Amyloid Precursor/Presenilin 1
TGF-β1	Transforming Growth Factor Beta 1
cGAS-STING	cGAS-STING Pathway
CD206	Cluster of Differentiation 206
TBK1/IRF3	TBK1/Interferon Reg. Factor 3
NLRP3	NOD-Like Receptor protein 3
HMGB1	High Mobility Group Box 1
MCAO	Middle Cerebral Artery Occlusion
HPA Axis	Hypothalamic-Pituitary-Adrenal Axis
BV-2	Brain Ventricular 2
HO-1	Heme Oxygenase 1
ALS	Amyotrophic Lateral Sclerosis
6-OHDA	6-Hydroxydopamine
GSH/GSSG	Reduced/Oxidized Glutathione
MDA	Malondialdehyde
SOD	Superoxide Dismutase
GSH-PX	Glutathione Peroxidase
PC12	Pheochromocytoma Cell Line 12
CAT	Catalase
SN	Substantia Nigra
EX527	Sirtinol II
SH-SY5Y	SH-SY5Y
BDNF	Brain-Derived Neurotrophic Factor
AMPA	AMPA Receptor
RAGE	Receptor for Advanced Glycation End Products
NQO1	NAD(P)H Quinone Dehydrogenase 1
CPZ	Cuprizone
MBP	Myelin Basic Protein
BMDM	Bone Marrow-Derived Macrophages
SCI	Spinal Cord Injury
mTOR	Mechanistic Target of Rapamycin
IAPS/XIAP	Inhibitors of Apoptosis Proteins
NFT	Neurofibrillary Tangles
JNK/MAPK	JNK/Mitogen-Activated Protein Kinase
GSK-3β	Glycogen Synthase Kinase 3 Beta
Aβ	Amyloid Beta Peptide
DG/CA3	Dentate Gyrus/Cornu Ammonis 3
TH	Tyrosine Hydroxylase
CUMS/CMS	Chronic Mild Stress
HIBD	Hypoxic-Ischemic Brain Damage
OGD/R	Oxygen-Glucose Deprivation
CMA	Chaperone-Mediated Autophagy
LC3	Light Chain 3
CREB	cAMP Response Element-Binding
UPR	Unfolded Protein Response
CHOP/ATF4	CHOP/ATF4
PUFA	Polyunsaturated Fatty Acids

## References

[1] Vujosevic S., Limoli C., Kozak I. (2025). Hallmarks of Aging in Age-Related Macular Degeneration and Age-Related Neurological Disorders: Novel Insights into Common Mechanisms and Clinical Relevance. EYE.

[2] Wang S., Jiang Y., Yang A., Meng F., Zhang J. (2024). The Expanding Burden of Neurodegenerative Diseases: An Unmet Medical and Social Need. Aging Dis..

[3] Hacker K. (2024). The Burden of Chronic Disease. Mayo Clin. Proc. Innov. Qual. Outcomes.

[4] Gorman A.M. (2008). Neuronal Cell Death in Neurodegenerative Diseases: Recurring Themes around Protein Handling. J. Cell. Mol. Med..

[5] Moujalled D., Strasser A., Liddell J.R. (2021). Molecular Mechanisms of Cell Death in Neurological Diseases. Cell Death Differ..

[6] Chi H., Chang H.-Y., Sang T.-K. (2018). Neuronal Cell Death Mechanisms in Major Neurodegenerative Diseases. Int. J. Mol. Sci..

[7] Fricker M., Tolkovsky A.M., Borutaite V., Coleman M., Brown G.C. (2018). Neuronal Cell Death. Physiol. Rev..

[8] Karvandi M.S., Sheikhzadeh Hesari F., Aref A.R., Mahdavi M. (2023). The Neuroprotective Effects of Targeting Key Factors of Neuronal Cell Death in Neurodegenerative Diseases: The Role of ER Stress, Oxidative Stress, and Neuroinflammation. Front. Cell. Neurosci..

[9] van Dam L., Dansen T.B. (2020). Cross-Talk between Redox Signalling and Protein Aggregation. Biochem. Soc. Trans..

[10] Liu N., Liu Y., Wang Y., Feng C., Piao M., Liu M. (2025). Oxidative Cell Death in the Central Nervous System: Mechanisms and Therapeutic Strategies. Front. Cell Dev. Biol..

[11] Lei L., Yuan J., Dai Z., Xiang S., Tu Q., Cui X., Zhai S., Chen X., He Z., Fang B. (2024). Targeting the Labile Iron Pool with Engineered DFO Nanosheets to Inhibit Ferroptosis for Parkinson’s Disease Therapy. Adv. Mater..

[12] Shi F.-D., Yong V.W. (2025). Neuroinflammation across Neurological Diseases. Science.

[13] Zhang W., Xiao D., Mao Q., Xia H. (2023). Role of Neuroinflammation in Neurodegeneration Development. Signal Transduct. Target. Ther..

[14] Lindholm D., Wootz H., Korhonen L. (2006). ER Stress and Neurodegenerative Diseases. Cell Death Differ..

[15] Hasan S.-A.-M., James A.W., Fazili F.M., Tarabishi S., Sheikh N.M., Shah Z.A. (2024). Endoplasmic Reticulum Stress in Neurodegenerative Diseases. J. Dement. Alzheimers Dis..

[16] Palmer J.E., Wilson N., Son S.M., Obrocki P., Wrobel L., Rob M., Takla M., Korolchuk V.I., Rubinsztein D.C. (2025). Autophagy, Aging, and Age-Related Neurodegeneration. Neuron.

[17] Menzies F.M., Fleming A., Caricasole A., Bento C.F., Andrews S.P., Ashkenazi A., Füllgrabe J., Jackson A., Jimenez Sanchez M., Karabiyik C. (2017). Autophagy and Neurodegeneration: Pathogenic Mechanisms and Therapeutic Opportunities. Neuron.

[18] Samanta S., Chakraborty S., Bagchi D. (2024). Pathogenesis of Neurodegenerative Diseases and the Protective Role of Natural Bioactive Components. J. Am. Nutr. Assoc..

[19] Ma H., He X., Yang Y., Li M., Hao D., Jia Z. (2011). The Genus Epimedium: An Ethnopharmacological and Phytochemical Review. J. Ethnopharmacol..

[20] Li C., Li Q., Mei Q., Lu T. (2015). Pharmacological Effects and Pharmacokinetic Properties of Icariin, the Major Bioactive Component in Herba Epimedii. Life Sci..

[21] Ding C. (1990). Determination of icarim in luohan jindan oral liquid by thin-layer chromatography. Zhongguo Zhong Yao Za Zhi.

[22] Bilia A.R., Ballerini R., Qu L., Wang M. (2025). Traditional Chinese Herbal Medicine in European Union: State of Art, Challenges, and Future Perspectives Focusing on Italian Market. Chin. Herb. Med..

[23] Wang S., Ma J., Zeng Y., Zhou G., Wang Y., Zhou W., Sun X., Wu M. (2021). Icariin, an up-and-Coming Bioactive Compound against Neurological Diseases: Network Pharmacology-Based Study and Literature Review. Drug Des. Devel. Ther..

[24] Li S., He M., He Y., Jin T., Chen J., Peng J., Hu W., He F. (2025). Icariin Supplementation Alleviates Cognitive Impairment Induced by D-Galactose via Modulation of the Gut-Brain Axis. J. Agric. Food Chem..

[25] Lu D.-S., Chen C., Zheng Y.-X., Li D.-D., Wang G.-Q., Liu J., Shi J., Zhang F. (2018). Combination Treatment of Icariin and L-DOPA against 6-OHDA-Lesioned Dopamine Neurotoxicity. Front. Mol. Neurosci..

[26] Zheng X.X., Li Y.C., Yang K.L., He Z.X., Wang Z.L., Wang X., Jing H.L., Cao Y.J. (2021). Icariin Reduces Glu-Induced Excitatory Neurotoxicity via Antioxidative and Antiapoptotic Pathways in SH-SY5Y Cells. Phytother. Res..

[27] Wang M., Yang X., Zhou Q., Guo Y., Chen Y., Song L., Yang J., Li L., Luo L. (2022). Neuroprotective Mechanism of Icariin on Hypoxic Ischemic Brain Damage in Neonatal Mice. Oxid. Med. Cell. Longev..

[28] Li L.R., Sethi G., Zhang X., Liu C.L., Huang Y., Liu Q., Ren B.X., Tang F.R. (2022). The Neuroprotective Effects of Icariin on Ageing, Various Neurological, Neuropsychiatric Disorders, and Brain Injury Induced by Radiation Exposure. Aging.

[29] Yan F., Liu J., Chen M.-X., Zhang Y., Wei S.-J., Jin H., Nie J., Fu X.-L., Shi J.-S., Zhou S.-Y. (2023). Icariin Ameliorates Memory Deficits through Regulating Brain Insulin Signaling and Glucose Transporters in 3×Tg-AD Mice. Neural Regen. Res..

[30] Xu C., Huang X., Tong Y., Feng X., Wang Y., Wang C., Jiang Y. (2020). Icariin Modulates the sirtuin/NF-κB Pathway and Exerts Anti-aging Effects in Human Lung Fibroblasts. Mol. Med. Rep..

[31] Zhu H.-R., Wang Z.-Y., Zhu X.-L., Wu X.-X., Li E.-G., Xu Y. (2010). Icariin Protects against Brain Injury by Enhancing SIRT1-Dependent PGC-1alpha Expression in Experimental Stroke. Neuropharmacology.

[32] Ni T., Lin N., Huang X., Lu W., Sun Z., Zhang J., Lin H., Chi J., Guo H. (2020). Icariin Ameliorates Diabetic Cardiomyopathy through Apelin/Sirt3 Signalling to Improve Mitochondrial Dysfunction. Front. Pharmacol..

[33] Li H., Zhang X., Qi X., Zhu X., Cheng L. (2019). Icariin Inhibits Endoplasmic Reticulum Stress-Induced Neuronal Apoptosis after Spinal Cord Injury through Modulating the PI3K/AKT Signaling Pathway. Int. J. Biol. Sci..

[34] Dai M., Chen B., Wang X., Gao C., Yu H. (2021). Icariin Enhance Mild Hypothermia-Induced Neuroprotection via Inhibiting the Activation of NF-κB in Experimental Ischemic Stroke. Metab. Brain Dis..

[35] Chen F.-J., Liu B., Wu Q., Liu J., Xu Y.-Y., Zhou S.-Y., Shi J.-S. (2019). Icariin Delays Brain Aging in Senescence-Accelerated Mouse Prone 8 (SAMP8) Model via Inhibiting Autophagy. J. Pharmacol. Exp. Ther..

[36] Yang Y., Fu Y., Qin Z., Pei H., Zhai L., Guan Q., Wu S., Shen H. (2023). Icariin Improves Cognitive Impairment by Inhibiting Ferroptosis of Nerve Cells. Aging.

[37] Rana J.N., Mumtaz S. (2025). Prunin: An Emerging Anticancer Flavonoid. Int. J. Mol. Sci..

[38] Zhang H.-W., Hu J.-J., Fu R.-Q., Liu X., Zhang Y.-H., Li J., Liu L., Li Y.-N., Deng Q., Luo Q.-S. (2018). Flavonoids Inhibit Cell Proliferation and Induce Apoptosis and Autophagy through Downregulation of PI3Kγ Mediated PI3K/AKT/mTOR/p70S6K/ULK Signaling Pathway in Human Breast Cancer Cells. Sci. Rep..

[39] Brusselmans K., Vrolix R., Verhoeven G., Swinnen J.V. (2005). Induction of Cancer Cell Apoptosis by Flavonoids Is Associated with Their Ability to Inhibit Fatty Acid Synthase Activity. J. Biol. Chem..

[40] Jomova K., Alomar S.Y., Valko R., Liska J., Nepovimova E., Kuca K., Valko M. (2025). Flavonoids and Their Role in Oxidative Stress, Inflammation, and Human Diseases. Chem. Biol. Interact..

[41] Kruszka J., Martyński J., Szewczyk-Golec K., Woźniak A., Nuszkiewicz J. (2025). The Role of Selected Flavonoids in Modulating Neuroinflammation in Alzheimer’s Disease: Mechanisms and Therapeutic Potential. Brain Sci..

[42] Al-Khayri J.M., Sahana G.R., Nagella P., Joseph B.V., Alessa F.M., Al-Mssallem M.Q. (2022). Flavonoids as Potential Anti-Inflammatory Molecules: A Review. Molecules.

[43] Cotelle N. (2001). Role of Flavonoids in Oxidative Stress. Curr. Top. Med. Chem..

[44] Leonardo C.C., Doré S. (2011). Dietary Flavonoids Are Neuroprotective through Nrf2-Coordinated Induction of Endogenous Cytoprotective Proteins. Nutr. Neurosci..

[45] Habtemariam S. (2019). The Nrf2/HO-1 Axis as Targets for Flavanones: Neuroprotection by Pinocembrin, Naringenin, and Eriodictyol. Oxid. Med. Cell. Longev..

[46] Fanaro G.B., Marques M.R., Calaza K.d.C., Brito R., Pessoni A.M., Mendonça H.R., Lemos D.E.d.A., de Brito Alves J.L., de Souza E.L., Cavalcanti Neto M.P. (2023). New Insights on Dietary Polyphenols for the Management of Oxidative Stress and Neuroinflammation in Diabetic Retinopathy. Antioxidants.

[47] Skibola C.F., Smith M.T. (2000). Potential Health Impacts of Excessive Flavonoid Intake. Free Radic. Biol. Med..

[48] Liu F.-Y., Ding D.-N., Wang Y.-R., Liu S.-X., Peng C., Shen F., Zhu X.-Y., Li C., Tang L.-P., Han F.-J. (2023). Icariin as a Potential Anticancer Agent: A Review of Its Biological Effects on Various Cancers. Front. Pharmacol..

[49] Chen Y., Wang J., Jia X., Tan X., Hu M. (2011). Role of Intestinal Hydrolase in the Absorption of Prenylated Flavonoids Present in Yinyanghuo. Molecules.

[50] Xu S., Yu J., Zhan J., Yang L., Guo L., Xu Y. (2017). Pharmacokinetics, Tissue Distribution, and Metabolism Study of Icariin in Rat. Biomed Res. Int..

[51] Cheng S., Qiu F., Wang S., He J. (2007). HPLC Analysis and Pharmacokinetics of Icariin in Rats. J. Sep. Sci..

[52] Jin J., Wang H., Hua X., Chen D., Huang C., Chen Z. (2019). An Outline for the Pharmacological Effect of Icariin in the Nervous System. Eur. J. Pharmacol..

[53] Liu J., Lou Y.-J. (2004). Determination of Icariin and Metabolites in Rat Serum by Capillary Zone Electrophoresis: Rat Pharmacokinetic Studies after Administration of Icariin. J. Pharm. Biomed. Anal..

[54] Zhang Y., Wang Q.-S., Cui Y.-L., Meng F.-C., Lin K.-M. (2012). Changes in the Intestinal Absorption Mechanism of Icariin in the Nanocavities of Cyclodextrins. Int. J. Nanomed..

[55] Zhou J., Chen Y., Wang Y., Gao X., Qu D., Liu C. (2013). A Comparative Study on the Metabolism of Epimedium Koreanum Nakai-Prenylated Flavonoids in Rats by an Intestinal Enzyme (lactase Phlorizin Hydrolase) and Intestinal Flora. Molecules.

[56] Zhao H., Fan M., Fan L., Sun J., Guo D. (2010). Liquid Chromatography-Tandem Mass Spectrometry Analysis of Metabolites in Rats after Administration of Prenylflavonoids from Epimediums. J. Chromatogr. B Analyt. Technol. Biomed. Life Sci..

[57] Qian Q., Li S.-L., Sun E., Zhang K.-R., Tan X.-B., Wei Y.-J., Fan H.-W., Cui L., Jia X.-B. (2012). Metabolite Profiles of Icariin in Rat Plasma by Ultra-Fast Liquid Chromatography Coupled to Triple-Quadrupole/time-of-Flight Mass Spectrometry. J. Pharm. Biomed. Anal..

[58] Cheng T., Sheng T., Yi Y., Zhang T., Han H. (2016). Metabolism Profiles of Icariin in Rats Using Ultra-High Performance Liquid Chromatography Coupled with Quadrupole Time-of-Flight Tandem Mass Spectrometry and in Vitro Enzymatic Study. J. Chromatogr. B Analyt. Technol. Biomed. Life Sci..

[59] Cheng T., Zhang Y., Zhang T., Lu L., Ding Y., Zhao Y. (2015). Comparative Pharmacokinetics Study of Icariin and Icariside II in Rats. Molecules.

[60] Yang W., Yu X.-C., Chen X.-Y., Zhang L., Lu C.-T., Zhao Y.-Z. (2012). Pharmacokinetics and Tissue Distribution Profile of Icariin Propylene Glycol-Liposome Intraperitoneal Injection in Mice: Icariin Liposome Pharmacokinetics and Tissue Distribution. J. Pharm. Pharmacol..

[61] Shi M., Kan H., Tang Y., Tian L., Guo X., Chen W., Geng J., Zong Y., Bi Y., He Z. (2025). Icariin Ameliorates Cyclophosphamide-Induced Renal Encephalopathy by Modulating the NF-κB and Keap1-Nrf2 Signaling Pathways. Int. J. Mol. Sci..

[62] Wang Y., Yang W., Zuo H., Zeng S., Liu X., Cao F., Yang H., Gao S., Tian M., Gao X. (2025). Icariin Interacts with IGFBP3 to Alleviate Diabetic Cataract through PI3K/AKT Signaling Pathway. iScience.

[63] Liu L., Zhao Z., Lu L., Liu J., Sun J., Wu X., Dong J. (2019). Icariin and Icaritin Ameliorated Hippocampus Neuroinflammation via Inhibiting HMGB1-Related pro-Inflammatory Signals in Lipopolysaccharide-Induced Inflammation Model in C57BL/6 J Mice. Int. Immunopharmacol..

[64] Jia G., Zhang Y., Li W., Dai H. (2019). Neuroprotective Role of Icariin in Experimental Spinal Cord Injury via Its Antioxidant, Anti-neuroinflammatory and Anti-apoptotic Properties. Mol. Med. Rep..

[65] Zeng R., Wang X., Zhou Q., Fu X., Wu Q., Lu Y., Shi J., Klaunig J.E., Zhou S. (2019). Icariin Protects Rotenone-Induced Neurotoxicity through Induction of SIRT3. Toxicol. Appl. Pharmacol..

[66] Wang C., Wang X., Xu L., Cheng Y. (2019). Neuroprotective Activity of Icariin against Hypoxic-Ischemic Brain Injury in Neonatal Rats. Int. J. Pharmacol..

[67] Wu J., Qu J.-Q., Zhou Y.-J., Zhou Y.-J., Li Y.-Y., Huang N.-Q., Deng C.-M., Luo Y. (2020). Icariin Improves Cognitive Deficits by Reducing the Deposition of β-Amyloid Peptide and Inhibition of Neurons Apoptosis in SAMP8 Mice. Neuroreport.

[68] Zheng J., Du M., Ye W., Xie J., Zhang P., Huang C., Lin H. (2025). Editorial: Molecular Mechanisms and Therapeutic Strategies in Inflammation. Front. Immunol..

[69] Wei Z., Wang M., Hong M., Diao S., Liu A., Huang Y., Yu Q., Peng Z. (2016). Icariin Exerts Estrogen-like Activity in Ameliorating EAE via Mediating Estrogen Receptor β, Modulating HPA Function and Glucocorticoid Receptor Expression. Am. J. Transl. Res..

[70] Cong H., Zhang M., Chang H., Du L., Zhang X., Yin L. (2020). Icariin Ameliorates the Progression of Experimental Autoimmune Encephalomyelitis by down-Regulating the Major Inflammatory Signal Pathways in a Mouse Relapse-Remission Model of Multiple Sclerosis. Eur. J. Pharmacol..

[71] Wang G., Li X., Li N., Wang X., He S., Li W., Fan W., Li R., Liu J., Hou S. (2022). Icariin Alleviates Uveitis by Targeting Peroxiredoxin 3 to Modulate Retinal Microglia M1/M2 Phenotypic Polarization. Redox Biol..

[72] Shen R., Deng W., Li C., Zeng G. (2015). A Natural Flavonoid Glucoside Icariin Inhibits Th1 and Th17 Cell Differentiation and Ameliorates Experimental Autoimmune Encephalomyelitis. Int. Immunopharmacol..

[73] Pokkula S., Thakur S.R. (2021). Icariin Ameliorates Partial Sciatic Nerve Ligation Induced Neuropathic Pain in Rats: An Evidence of in Silico and in Vivo Studies. J. Pharm. Pharmacol..

[74] Lin Z., Zhong R., Xu Y., Wu Y., Ru C. (2024). Shell-Core Structured Nanofibers Mediate Staged Anti-Inflammatory and pro-Neurogenic Activities to Repair Peripheral Nerve. Mater. Res. Express.

[75] Wang L., Peng G., Chen L., Guo M., Wang B., Zhang Y., Zhou J., Zhong M., Ye J. (2023). Icariin Reduces Cognitive Dysfunction Induced by Surgical Trauma in Aged Rats by Inhibiting Hippocampal Neuroinflammation. Front. Behav. Neurosci..

[76] Lu F., Li L., Zheng B., Wang C., Liu Z., Huang X., Song L., Ding C., Li Y. (2025). Icariin Alleviates Cognitive Dysfunction by Reducing Neuroinflammation via the cGAS-STING Pathway. J. Ethnopharmacol..

[77] Zhang Z.-Y., Li C., Zug C., Schluesener H.J. (2014). Icariin Ameliorates Neuropathological Changes, TGF-β1 Accumulation and Behavioral Deficits in a Mouse Model of Cerebral Amyloidosis. PLoS ONE.

[78] Li H., Xiao Q., Zhu L., Kang J., Zhan Q., Peng W. (2025). Targeting Ceramide-Induced Microglial Pyroptosis: Icariin Is a Promising Therapy for Alzheimer’s Disease. J. Pharm. Anal..

[79] Wang J., Liu Y., Wu Y., Yang K., Yang K., Yan L., Feng L. (2023). Anti-Inflammatory Effects of Icariin in the Acute and Chronic Phases of the Mouse Pilocarpine Model of Epilepsy. Eur. J. Pharmacol..

[80] Zheng J., Liao Y., Xu Y., Mo Z. (2022). Icariin Attenuates Ischaemic Stroke through Suppressing Inflammation Mediated by Endoplasmic Reticulum Stress Signalling Pathway in Rats. Clin. Exp. Pharmacol. Physiol..

[81] Li T., Li S., Xiong Y., Li X., Ma C., Guan Z., Yang L. (2024). Binary Nano-Inhalant Formulation of Icariin Enhances Cognitive Function in Vascular Dementia via BDNF/TrkB Signaling and Anti-Inflammatory Effects. Neurochem. Res..

[82] Zheng Y., Zhu G., He J., Wang G., Li D., Zhang F. (2019). Icariin Targets Nrf2 Signaling to Inhibit Microglia-Mediated Neuroinflammation. Int. Immunopharmacol..

[83] Zhang B., Wang G., He J., Yang Q., Li D., Li J., Zhang F. (2019). Icariin Attenuates Neuroinflammation and Exerts Dopamine Neuroprotection via an Nrf2-Dependent Manner. J. Neuroinflammation.

[84] Li H., Zhang X., Zhu X., Qi X., Lin K., Cheng L. (2018). The Effects of Icariin on Enhancing Motor Recovery through Attenuating pro-Inflammatory Factors and Oxidative Stress via Mitochondrial Apoptotic Pathway in the Mice Model of Spinal Cord Injury. Front. Physiol..

[85] Sharma S., Mehan S., Khan Z., Gupta G.D., Narula A.S. (2024). Icariin Prevents Methylmercury-Induced Experimental Neurotoxicity: Evidence from Cerebrospinal Fluid, Blood Plasma, Brain Samples, and in-Silico Investigations. Heliyon.

[86] Xu N., Huang F., Jian C., Qin L., Lu F., Wang Y., Zhang Z., Zhang Q. (2019). Neuroprotective Effect of Salidroside against Central Nervous System Inflammation-Induced Cognitive Deficits: A Pivotal Role of Sirtuin 1-Dependent Nrf-2/HO-1/NF-κB Pathway: Salidroside Alleviates Cognitive Deficits. Phytother. Res..

[87] Dong J., Zhang X., Wang S., Xu C., Gao M., Liu S., Li X., Cheng N., Han Y., Wang X. (2020). Thymoquinone Prevents Dopaminergic Neurodegeneration by Attenuating Oxidative Stress via the Nrf2/ARE Pathway. Front. Pharmacol..

[88] Zhang W.-D., Li N., Du Z.-R., Zhang M., Chen S., Chen W.-F. (2021). IGF-1 Receptor Is Involved in the Regulatory Effects of Icariin and Icaritin in Astrocytes under Basal Conditions and after an Inflammatory Challenge. Eur. J. Pharmacol..

[89] Liu B., Xu C., Wu X., Liu F., Du Y., Sun J., Tao J., Dong J. (2015). Icariin Exerts an Antidepressant Effect in an Unpredictable Chronic Mild Stress Model of Depression in Rats and Is Associated with the Regulation of Hippocampal Neuroinflammation. Neuroscience.

[90] Xiong D., Deng Y., Huang B., Yin C., Liu B., Shi J., Gong Q. (2016). Icariin Attenuates Cerebral Ischemia-Reperfusion Injury through Inhibition of Inflammatory Response Mediated by NF-κB, PPARα and PPARγ in Rats. Int. Immunopharmacol..

[91] Mo Z.-T., Li W.-N., Zhai Y.-R., Gao S.-Y. (2017). The Effects of Icariin on the Expression of HIF-1α, HSP-60 and HSP-70 in PC12 Cells Suffered from Oxygen-Glucose Deprivation-Induced Injury. Pharm. Biol..

[92] Song L.-J., Han Q.-X., Ding Z.-B., Liu K., Zhang X.-X., Guo M.-F., Ma D., Wang Q., Xiao B.-G., Ma C.-G. (2024). Icariin Ameliorates the Cuprizone-Induced Demyelination Associated with Antioxidation and Anti-Inflammation. Inflammopharmacology.

[93] Chen S., Zou R., Si J., Shi Q., Zhang L., Kang L., Ni J., Sha D. (2024). Icariin Inhibits Apoptosis in OGD-Induced Neurons by Regulating M2 Pyruvate Kinase. IBRO Neurosci. Rep..

[94] Sies H., Jones D.P. (2020). Reactive Oxygen Species (ROS) as Pleiotropic Physiological Signalling Agents. Nat. Rev. Mol. Cell Biol..

[95] Stewart V.C., Heales S.J.R. (2003). Nitric Oxide-Induced Mitochondrial Dysfunction: Implications for Neurodegeneration. Free Radic. Biol. Med..

[96] de Freitas Azevedo-Repossi R., Brito R., Cossenza M., Dos Santos-Rodrigues A., Ferreira G.C., Petrs-Silva H., Calaza K.C., Fragel-Madeira L. (2025). Reactive Oxygen Species Regulation across Retinitis Pigmentosa Animal Models: A 25-Year Systematized Review. Mol. Neurobiol..

[97] Sies H. (2015). Oxidative Stress: A Concept in Redox Biology and Medicine. Redox Biol..

[98] Tempone M.H., Borges-Martins V.P., César F., Alexandrino-Mattos D.P., de Figueiredo C.S., Raony Í., Dos Santos A.A., Duarte-Silva A.T., Dias M.S., Freitas H.R. (2024). The Healthy and Diseased Retina Seen through Neuron-Glia Interactions. Int. J. Mol. Sci..

[99] Baird L., Yamamoto M. (2020). The Molecular Mechanisms Regulating the KEAP1-NRF2 Pathway. Mol. Cell. Biol..

[100] Joo H., Bae J., Lee J.-S., Bang Y., Lee B.-J., Park J.-W., Lee K., Cho J.-H., Bu Y. (2019). Icariin Improves Functional Behavior in a Mouse Model of Traumatic Brain Injury and Promotes Synaptic Plasticity Markers. Planta Med..

[101] Ning K., Gao R. (2023). Icariin Protects Cerebral Neural Cells from Ischemia-reperfusion Injury in an in Vitro Model by Lowering ROS Production and Intracellular Calcium Concentration. Exp. Ther. Med..

[102] Guo Y., Cai Y., Zhang X. (2020). Icariin Ameliorates the Cognitive Function in an Epilepsy Neonatal Rat Model by Blocking the GluR2/ERK I/II Pathway. Folia Neuropathol..

[103] Xu R.-X., Wu Q., Luo Y., Gong Q.-H., Yu L.-M., Huang X.-N., Sun A.-S., Shi J.-S. (2009). Protective Effects of Icariin on Cognitive Deficits Induced by Chronic Cerebral Hypoperfusion in Rats. Clin. Exp. Pharmacol. Physiol..

[104] Zheng M., Qu L., Lou Y. (2008). Effects of Icariin Combined with Panax Notoginseng Saponins on Ischemia Reperfusion-Induced Cognitive Impairments Related with Oxidative Stress and CA1 of Hippocampal Neurons in Rat. Phytother. Res..

[105] Nwachukwu K., Rhoads E., Meek S., Bardi M. (2021). Back to Nature: Herbal Treatment, Environmental Enrichment, and Social Play Can Protect against Unpredictable Chronic Stress in Long-Evans Rats (*Rattus norvegicus*). Psychopharmacology.

[106] Zhu L., Li D., Chen C., Wang G., Shi J., Zhang F. (2019). Activation of Nrf2 Signaling by Icariin Protects against 6-OHDA-Induced Neurotoxicity. Biotechnol. Appl. Biochem..

[107] Li W.-W., Gao X.-M., Wang X.-M., Guo H., Zhang B.-L. (2011). Icariin Inhibits Hydrogen Peroxide-Induced Toxicity through Inhibition of Phosphorylation of JNK/p38 MAPK and p53 Activity. Mutat. Res..

[108] Zhang L., Huang S., Chen Y., Wang Z., Li E., Xu Y. (2010). Icariin Inhibits Hydrogen Peroxide-Mediated Cytotoxicity by up-Regulating Sirtuin Type 1-Dependent Catalase and Peroxiredoxin. Basic Clin. Pharmacol. Toxicol..

[109] Zhao S.-Y., Liao L.-X., Tu P.-F., Li W.-W., Zeng K.-W. (2019). Icariin Inhibits AGE-Induced Injury in PC12 Cells by Directly Targeting Apoptosis Regulator Bax. Oxid. Med. Cell. Longev..

[110] Yuan P., Chen W., Wang X., Li L., Peng Z., Mu S., You M., Xu H. (2024). RAGE: A Potential Target for Epimedium’s Anti-Neuroinflammation Role in Vascular Dementia-Insights from Network Pharmacology and Molecular Simulation. J. Biomol. Struct. Dyn..

[111] He X.-L., Zhou W.-Q., Bi M.-G., Du G.-H. (2010). Neuroprotective Effects of Icariin on Memory Impairment and Neurochemical Deficits in Senescence-Accelerated Mouse Prone 8 (SAMP8) Mice. Brain Res..

[112] Zhang Y., Huang N., Lu H., Huang J., Jin H., Shi J., Jin F. (2020). Icariin Protects against Sodium Azide-Induced Neurotoxicity by Activating the PI3K/Akt/GSK-3β Signaling Pathway. PeerJ.

[113] Wang H., Tang Q., Xue Y., Gao X., Zhang Y. (2023). Discovery of Drug Lead Compounds for Anti-Alzheimer’s Disease on the Basis of Synaptic Plasticity. Heliyon.

[114] Song Y.-X., Miao J.-Y., Qiang M., He R.-Q., Wang X.-M., Li W.-W. (2016). Icariin Protects SH-SY5Y Cells from Formaldehyde-Induced Injury through Suppression of Tau Phosphorylation. Chin. J. Integr. Med..

[115] Zhang Y., Kong W.-N., Chai X.-Q. (2018). Compound of Icariin, Astragalus, and Puerarin Mitigates Iron Overload in the Cerebral Cortex of Alzheimer’s Disease Mice. Neural Regen. Res..

[116] Sha D., Li L., Ye L., Liu R., Xu Y. (2009). Icariin Inhibits Neurotoxicity of Beta-Amyloid by Upregulating Cocaine-Regulated and Amphetamine-Regulated Transcripts. Neuroreport.

[117] Zhou N., Tang Y., Keep R.F., Ma X., Xiang J. (2014). Antioxidative Effects of Panax Notoginseng Saponins in Brain Cells. Phytomedicine.

[118] Lei X., Wen D., Huang Z., Li X., Tang L., Zhu Y., Guo Z. (2025). Icariin Attenuates Oxidative Stress via SIRT1/PGC-1α Pathway in SAH Mice. Exp. Neurol..

[119] Rana J.N., Gul K., Mumtaz S. (2025). Isorhamnetin: Reviewing Recent Developments in Anticancer Mechanisms and Nanoformulation-Driven Delivery. Int. J. Mol. Sci..

[120] Sethi P., Mehan S., Khan Z., Maurya P.K., Kumar N., Kumar A., Tiwari A., Sharma T., Das Gupta G., Narula A.S. (2025). The SIRT-1/Nrf2/HO-1 Axis: Guardians of Neuronal Health in Neurological Disorders. Behav. Brain Res..

[121] Li X., Khan I., Xia W., Huang G., Liu L., Law B.Y.K., Yin L., Liao W., Leong W., Han R. (2021). Icariin Enhances Youth-like Features by Attenuating the Declined Gut Microbiota in the Aged Mice. Pharmacol. Res..

[122] Vitale I., Pietrocola F., Guilbaud E., Aaronson S.A., Abrams J.M., Adam D., Agostini M., Agostinis P., Alnemri E.S., Altucci L. (2023). Apoptotic Cell Death in Disease-Current Understanding of the NCCD 2023. Cell Death Differ..

[123] Mustafa M., Ahmad R., Tantry I.Q., Ahmad W., Siddiqui S., Alam M., Abbas K., Moinuddin, Hassan M.I., Habib S. (2024). Apoptosis: A Comprehensive Overview of Signaling Pathways, Morphological Changes, and Physiological Significance and Therapeutic Implications. Cells.

[124] Marivin A., Berthelet J., Plenchette S., Dubrez L. (2012). The Inhibitor of Apoptosis (IAPs) in Adaptive Response to Cellular Stress. Cells.

[125] Fan C., Yang Y., Liu Y., Jiang S., Di S., Hu W., Ma Z., Li T., Zhu Y., Xin Z. (2016). Icariin Displays Anticancer Activity against Human Esophageal Cancer Cells via Regulating Endoplasmic Reticulum Stress-Mediated Apoptotic Signaling. Sci. Rep..

[126] D’Arcy M.S. (2019). Cell Death: A Review of the Major Forms of Apoptosis, Necrosis and Autophagy. Cell Biol. Int..

[127] Sharma V.K., Singh T.G., Singh S., Garg N., Dhiman S. (2021). Apoptotic Pathways and Alzheimer’s Disease: Probing Therapeutic Potential. Neurochem. Res..

[128] Zeng K.-W., Ko H., Yang H.O., Wang X.-M. (2010). Icariin Attenuates β-Amyloid-Induced Neurotoxicity by Inhibition of Tau Protein Hyperphosphorylation in PC12 Cells. Neuropharmacology.

[129] Zhang D., Wang Z., Sheng C., Peng W., Hui S., Gong W., Chen S. (2015). Icariin Prevents Amyloid Beta-Induced Apoptosis via the PI3K/Akt Pathway in PC-12 Cells. Evid. Based. Complement. Alternat. Med..

[130] Li F., Zhang Y., Lu X., Shi J., Gong Q. (2019). Icariin Improves the Cognitive Function of APP/PS1 Mice via Suppressing Endoplasmic Reticulum Stress. Life Sci..

[131] Li F., Dong H.X., Gong Q.H., Wu Q., Jin F., Shi J.S. (2015). Icariin Decreases Both APP and Aβ Levels and Increases Neurogenesis in the Brain of Tg2576 Mice. Neuroscience.

[132] Sabry H.A., Zahra M.M. (2024). Icariin Attenuates Dopaminergic Neural Loss in Haloperidol-Induced Parkinsonism in Rats via GSK-3β and Tyrosine Hydroxylase Regulation Mechanism. J. Chem. Neuroanat..

[133] Chen W.-F., Wu L., Du Z.-R., Chen L., Xu A.-L., Chen X.-H., Teng J.-J., Wong M.-S. (2017). Neuroprotective Properties of Icariin in MPTP-Induced Mouse Model of Parkinson’s Disease: Involvement of PI3K/Akt and MEK/ERK Signaling Pathways. Phytomedicine.

[134] Liu B., Zhang H., Xu C., Yang G., Tao J., Huang J., Wu J., Duan X., Cao Y., Dong J. (2011). Neuroprotective Effects of Icariin on Corticosterone-Induced Apoptosis in Primary Cultured Rat Hippocampal Neurons. Brain Res..

[135] Zhang H., Liu B., Wu J., Xu C., Tao J., Duan X., Cao Y., Dong J. (2012). Icariin Inhibits Corticosterone-Induced Apoptosis in Hypothalamic Neurons via the PI3-K/Akt Signaling Pathway. Mol. Med. Rep..

[136] Shindel A.W., Xin Z.-C., Lin G., Fandel T.M., Huang Y.-C., Banie L., Breyer B.N., Garcia M.M., Lin C.-S., Lue T.F. (2010). Erectogenic and Neurotrophic Effects of Icariin, a Purified Extract of Horny Goat Weed (Epimedium Spp.) in Vitro and in Vivo. J. Sex. Med..

[137] Pan B., Xu L., Weng J., Wang Y., Ji H., Han B., Zhu X., Liu Y. (2022). Effects of Icariin on Alleviating Schizophrenia-like Symptoms by Regulating the miR-144-3p/ATP1B2/mTOR Signalling Pathway. Neurosci. Lett..

[138] Mo Z.-T., Liao Y.-L., Zheng J., Li W.-N. (2020). Icariin Protects Neurons from Endoplasmic Reticulum Stress-Induced Apoptosis after OGD/R Injury via Suppressing IRE1α-XBP1 Signaling Pathway. Life Sci..

[139] Wang M., Rong Y., Luo L. (2021). Neuroprotective Effects of Icariin in Neonatal Hypoxia-Ischemic Brain Damage via Its Anti-Apoptotic Property. Childs. Nerv. Syst..

[140] Wu C.-T., Chen M.-C., Liu S.-H., Yang T.-H., Long L.-H., Guan S.-S., Chen C.-M. (2021). Bioactive Flavonoids Icaritin and Icariin Protect against Cerebral Ischemia-Reperfusion-Associated Apoptosis and Extracellular Matrix Accumulation in an Ischemic Stroke Mouse Model. Biomedicines.

[141] Mo Z.-T., Li W.-N., Zhai Y.-R., Gong Q.-H. (2016). Icariin Attenuates OGD/R-Induced Autophagy via Bcl-2-Dependent Cross Talk between Apoptosis and Autophagy in PC12 Cells. Evid. Based. Complement. Alternat. Med..

[142] Yao X., Jing X., Guo J., Sun K., Deng Y., Zhang Y., Guo F., Ye Y. (2019). Icariin Protects Bone Marrow Mesenchymal Stem Cells against Iron Overload Induced Dysfunction through Mitochondrial Fusion and Fission, PI3K/AKT/mTOR and MAPK Pathways. Front. Pharmacol..

[143] Sadeghi A., Hami J., Razavi S., Esfandiary E., Hejazi Z. (2016). The Effect of Diabetes Mellitus on Apoptosis in Hippocampus: Cellular and Molecular Aspects. Int. J. Prev. Med..

[144] Bridge P.M., Ball D.J., Mackinnon S.E., Nakao Y., Brandt K., Hunter D.A., Hertl C. (1994). Nerve Crush Injuries--a Model for Axonotmesis. Exp. Neurol..

[145] Hannan J.L., Matsui H., Sopko N.A., Liu X., Weyne E., Albersen M., Watson J.W., Hoke A., Burnett A.L., Bivalacqua T.J. (2016). Caspase-3 Dependent Nitrergic Neuronal Apoptosis Following Cavernous Nerve Injury Is Mediated via RhoA and ROCK Activation in Major Pelvic Ganglion. Sci. Rep..

[146] Song G., Hu P., Song J., Liu J., Ruan Y. (2022). Molecular Pathogenesis and Treatment of Cavernous Nerve Injury-Induced Erectile Dysfunction: A Narrative Review. Front. Physiol..

[147] Wang H., Dong Z., Liu J., Zhu Z., Najafi M. (2023). Mechanisms of Cancer-Killing by Quercetin; A Review on Cell Death Mechanisms. Anticancer Agents Med. Chem..

[148] Mansuri M.L., Parihar P., Solanki I., Parihar M.S. (2014). Flavonoids in Modulation of Cell Survival Signalling Pathways. Genes Nutr..

[149] Yamamoto H., Matsui T. (2024). Molecular Mechanisms of Macroautophagy, Microautophagy, and Chaperone-Mediated Autophagy. J. Nippon Med. Sch..

[150] Liu S., Yao S., Yang H., Liu S., Wang Y. (2023). Autophagy: Regulator of Cell Death. Cell Death Dis..

[151] Kirchner P., Bourdenx M., Madrigal-Matute J., Tiano S., Diaz A., Bartholdy B.A., Will B., Cuervo A.M. (2019). Proteome-Wide Analysis of Chaperone-Mediated Autophagy Targeting Motifs. PLoS Biol..

[152] Zapatería B., Arias E. (2024). Aging, Cancer, and Autophagy: Connections and Therapeutic Perspectives. Front. Mol. Biosci..

[153] Eisenberg-Lerner A., Bialik S., Simon H.-U., Kimchi A. (2009). Life and Death Partners: Apoptosis, Autophagy and the Cross-Talk between Them. Cell Death Differ..

[154] Cecarini V., Bonfili L., Gogoi O., Lawrence S., Venanzi F.M., Azevedo V., Mancha-Agresti P., Drumond M.M., Rossi G., Berardi S. (2020). Neuroprotective Effects of p62(SQSTM1)-Engineered Lactic Acid Bacteria in Alzheimer’s Disease: A Pre-Clinical Study. Aging.

[155] Wang N., Wang H., Pan Q., Kang J., Liang Z., Zhang R. (2021). The Combination of β-Asarone and Icariin Inhibits Amyloid-β and Reverses Cognitive Deficits by Promoting Mitophagy in Models of Alzheimer’s Disease. Oxid. Med. Cell. Longev..

[156] Zhao H., Zhang S., Gong Z., Chen T., Guo H., Yang M., Wang L., Zhou X., Xie J., Li H. (2025). Icariin Mitigates Depressive-like Behavior in Rats by Regulating Mitochondrial Function. J. Ethnopharmacol..

[157] Jiang X., Chen L.-L., Lan Z., Xiong F., Xu X., Yin Y.-Y., Li P., Wang P. (2019). Icariin Ameliorates Amyloid Pathologies by Maintaining Homeostasis of Autophagic Systems in Aβ1-42-Injected Rats. Neurochem. Res..

[158] Filomeni G., De Zio D., Cecconi F. (2015). Oxidative Stress and Autophagy: The Clash between Damage and Metabolic Needs. Cell Death Differ..

[159] Levonen A.-L., Hill B.G., Kansanen E., Zhang J., Darley-Usmar V.M. (2014). Redox Regulation of Antioxidants, Autophagy, and the Response to Stress: Implications for Electrophile Therapeutics. Free Radic. Biol. Med..

[160] Gao Q. (2019). Oxidative Stress and Autophagy. Adv. Exp. Med. Biol..

[161] Tasca C.I., Dal-Cim T., Cimarosti H. (2015). In Vitro Oxygen-Glucose Deprivation to Study Ischemic Cell Death. Methods Mol. Biol..

[162] He Y., Mo Z., Xue Z., Fang Y. (2013). Establish a Flow Cytometric Method for Quantitative Detection of Beclin-1 Expression. Cytotechnology.

[163] Zong N., Li F., Deng Y., Shi J., Jin F., Gong Q. (2016). Icariin, a Major Constituent from Epimedium Brevicornum, Attenuates Ibotenic Acid-Induced Excitotoxicity in Rat Hippocampus. Behav. Brain Res..

[164] Zahra M.M., Ali E.H.A., Sabry H.A. (2024). Icariin Is a Potential Neurodegenerative Candidate against Ammonia–glutamate Excitotoxicity–oxidative Stress Pathway. J. Basic Appl. Zool..

[165] Wu C., Yang G., Pan Y., Wang L., Tu P., Zheng S., Guo Y., Ma Y. (2021). Icariin Promotes the Repair of PC12 Cells by Inhibiting Endoplasmic Reticulum Stress. BMC Complement. Med. Ther..

[166] Liu J., Liu L., Sun J., Luo Q., Yan C., Zhang H., Liu F., Wei Y., Dong J. (2019). Icariin Protects Hippocampal Neurons from Endoplasmic Reticulum Stress and NF-κB Mediated Apoptosis in Fetal Rat Hippocampal Neurons and Asthma Rats. Front. Pharmacol..

[167] Chen Y.-C., Zheng Q., Lu H., Zhuang Y.-X., Gong T.-T., Ma L.-F., Zhan Z.-J. (2023). Neuroprotective Flavonoids from Epimedium Brevicornu by Inhibition of Ferroptosis. Phytochem. Lett..

[168] Xiao J., Luo C., Li A., Cai F., Wang Y., Pan X., Xu L., Wang Z., Xing Z., Yu L. (2024). Icariin Inhibits Chondrocyte Ferroptosis and Alleviates Osteoarthritis by Enhancing the SLC7A11/GPX4 Signaling. Int. Immunopharmacol..

[169] Luo H., Zhang R. (2021). Icariin Enhances Cell Survival in Lipopolysaccharide-Induced Synoviocytes by Suppressing Ferroptosis via the Xc^−^/GPX4 Axis. Exp. Ther. Med..

[170] Kwon Y., Kim J.W., Jeoung J.A., Kim M.-S., Kang C. (2017). Autophagy Is pro-Senescence When Seen in Close-Up, but Anti-Senescence in Long-Shot. Mol. Cells.

[171] Azman K.F., Zakaria R. (2019). D-Galactose-Induced Accelerated Aging Model: An Overview. Biogerontology.

[172] Hu S.-S., Wang T.-Y., Ni L., Hu F.-X., Yue B.-W., Zheng Y., Wang T.-L., Kumar A., Wang Y.-Y., Wang J.-E. (2024). Icariin Ameliorates D-Galactose-Induced Cell Injury in Neuron-like PC12 Cells by Inhibiting MPTP Opening. Curr. Med. Sci..

[173] Wu B., Xiao Q., Zhu L., Tang H., Peng W. (2024). Icariin Targets p53 to Protect against Ceramide-Induced Neuronal Senescence: Implication in Alzheimer’s Disease. Free Radic. Biol. Med..

[174] Wu W.-L., Gong X.-X., Qin Z.-H., Wang Y. (2025). Molecular Mechanisms of Excitotoxicity and Their Relevance to the Pathogenesis of Neurodegenerative Diseases-an Update. Acta Pharmacol. Sin..

[175] Braakman I., Bulleid N.J. (2011). Protein Folding and Modification in the Mammalian Endoplasmic Reticulum. Annu. Rev. Biochem..

[176] Oakes S.A., Papa F.R. (2015). The Role of Endoplasmic Reticulum Stress in Human Pathology. Annu. Rev. Pathol..

[177] Victor P., Sarada D., Ramkumar K.M. (2021). Crosstalk between Endoplasmic Reticulum Stress and Oxidative Stress: Focus on Protein Disulfide Isomerase and Endoplasmic Reticulum Oxidase 1. Eur. J. Pharmacol..

[178] Zhai T., Wang B., Shi C., Zhang C., Shen J., Feng X., Gao F., Yang Y., Jia K., Zhao L. (2025). The Interplay between Endoplasmic Reticulum Stress and Ferroptosis in Neurological Diseases. Neurochem. Res..

[179] Lu Y., Zhou J., Wang H., Gao H., Ning E., Shao Z., Hao Y., Yang X. (2024). Endoplasmic Reticulum Stress-Mediated Apoptosis and Autophagy in Osteoarthritis: From Molecular Mechanisms to Therapeutic Applications. Cell Stress Chaperones.

[180] Berndt C., Alborzinia H., Amen V.S., Ayton S., Barayeu U., Bartelt A., Bayir H., Bebber C.M., Birsoy K., Böttcher J.P. (2024). Ferroptosis in Health and Disease. Redox Biol..

[181] Dixon S.J., Olzmann J.A. (2024). The Cell Biology of Ferroptosis. Nat. Rev. Mol. Cell Biol..

[182] Zhang F., Tao Y., Zhang Z., Guo X., An P., Shen Y., Wu Q., Yu Y., Wang F. (2012). Metalloreductase Steap3 Coordinates the Regulation of Iron Homeostasis and Inflammatory Responses. Haematologica.

[183] Tang D., Chen X., Kang R., Kroemer G. (2021). Ferroptosis: Molecular Mechanisms and Health Implications. Cell Res..

[184] Chen X., Yu C., Kang R., Kroemer G., Tang D. (2021). Cellular Degradation Systems in Ferroptosis. Cell Death Differ..

[185] Yang W.S., SriRamaratnam R., Welsch M.E., Shimada K., Skouta R., Viswanathan V.S., Cheah J.H., Clemons P.A., Shamji A.F., Clish C.B. (2014). Regulation of Ferroptotic Cancer Cell Death by GPX4. Cell.

[186] Sato H., Tamba M., Kuriyama-Matsumura K., Okuno S., Bannai S. (2000). Molecular Cloning and Expression of Human xCT, the Light Chain of Amino Acid Transport System Xc^−^. Antioxid. Redox Signal..

[187] Costa I., Barbosa D.J., Benfeito S., Silva V., Chavarria D., Borges F., Remião F., Silva R. (2023). Molecular Mechanisms of Ferroptosis and Their Involvement in Brain Diseases. Pharmacol. Ther..

[188] Dixon S.J., Lemberg K.M., Lamprecht M.R., Skouta R., Zaitsev E.M., Gleason C.E., Patel D.N., Bauer A.J., Cantley A.M., Yang W.S. (2012). Ferroptosis: An Iron-Dependent Form of Nonapoptotic Cell Death. Cell.

[189] Tian Y., Lu J., Hao X., Li H., Zhang G., Liu X., Li X., Zhao C., Kuang W., Chen D. (2020). FTH1 Inhibits Ferroptosis through Ferritinophagy in the 6-OHDA Model of Parkinson’s Disease. Neurotherapeutics.

[190] Venkatesh D., O’Brien N.A., Zandkarimi F., Tong D.R., Stokes M.E., Dunn D.E., Kengmana E.S., Aron A.T., Klein A.M., Csuka J.M. (2020). MDM2 and MDMX Promote Ferroptosis by PPARα-Mediated Lipid Remodeling. Genes Dev..

[191] Yan C.-Y., Gu X.-Y., Tan S.-Y., Mei A.-Y., Mao J.-H., Dai Y., Niu J., Li W.-X., Kurihara H., Li Y.-F. (2025). Lipid Peroxidation Inhibition by Icaritin and Its Glycosides as a Strategy to Combat Iron Overload-Induced Osteoporosis in Zebrafish. Food Res. Int..

[192] Fu B., Yu S., Chen S., Hu B. (2025). Icariin against Osteoporosis: A Review of Advances in Molecular Mechanisms to Biomedical Applications. Front. Pharmacol..

