# Supplementary material for: Icariin Against Neurodegeneration: A Focus in Cell Death Pathways
Source: Int J Mol Sci. 2026 Feb 27;27(5):2247. doi: 10.3390/ijms27052247 (PMC12984914; doi:10.3390/ijms27052247)
Supplement: Supplementary file 1 [file ijms-27-02247-s001.zip › ijms-4101204-supplementary.pdf]

Supplementary information

Icariin Against Neurodegeneration: A Focus in Cell Death Pathways

1. Methodology

1.1 Search Strategy Design

We use the search strategy CoCoPop (Condition, Context, Population) [135]. The Condition encompasses eight mechanisms of pathogenesis in the Nervous System: oxidative stress, inflammation, apoptosis, necrosis, autophagy, ferroptosis, excitotoxicity, and tumor. The Context was defined as the ICA intervention, while the Population corresponded to different experimental models of the nervous system. Thus, eight search strategies were developed, one for each condition, and adapted for the Medline/PubMed, Scopus, and Web of Science databases (Supplementary Table S1) until September 29th, 2025. After compiling the results and removing duplicates, the articles underwent an initial screening based on the reading of titles and abstracts, applying predefined inclusion and exclusion criteria (see next topic). The articles selected at this stage were then subjected to full-text analysis, in which the same eligibility criteria were again applied. Finally, data from the included studies were extracted, organized, and synthesized in a narrative review format (Supplementary Figure S1).

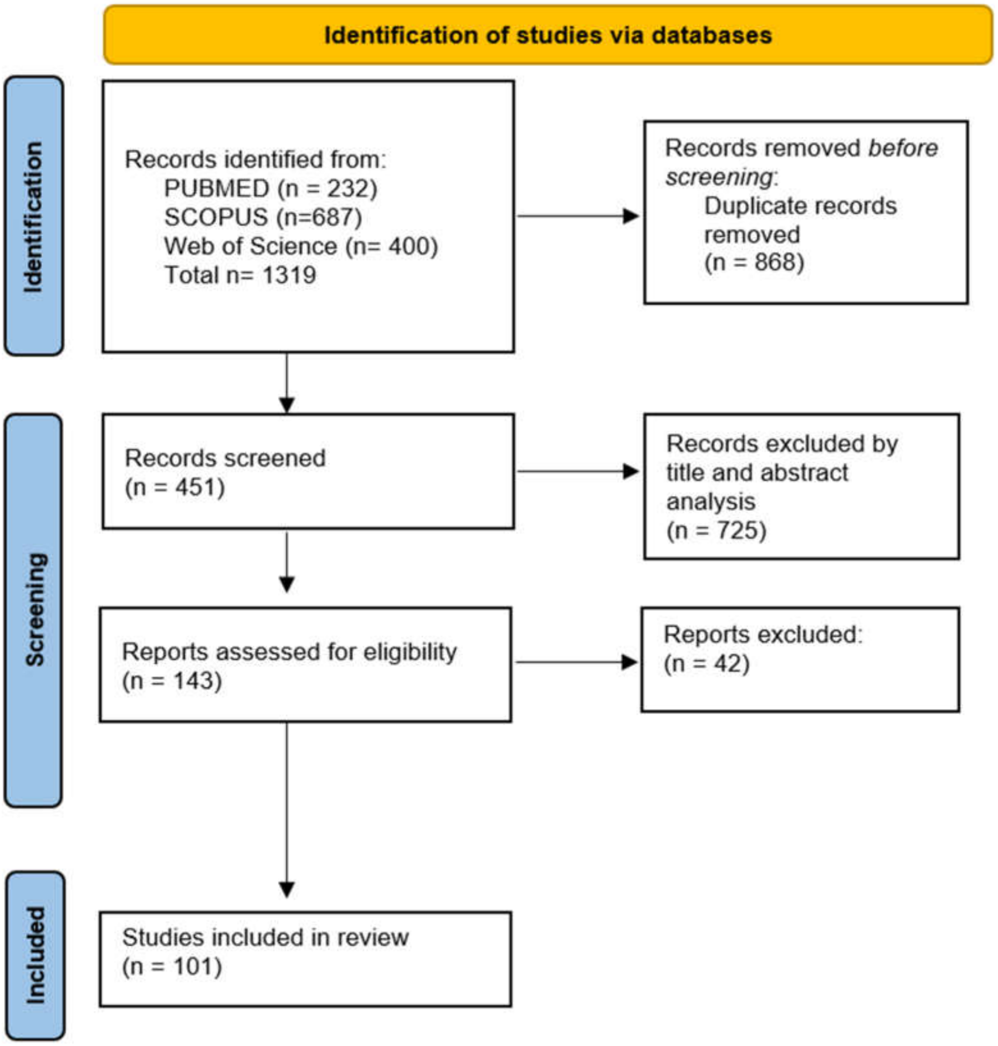

Supplementary Figure S1. Schematic flow diagram for Methodology of Screening for Narrative Review. Adaptation of PRISMA 2020 flow diagram for new systematic reviews, which included searches of databases and registers only. Source: [136].

Supplemental Table S1. Search keys for Pubmed/MEDLINE, Scopus, and Web of Science for each cell death type using the "CoCoPop" strategy. Results by database and number of results after removing duplicates for each cell death type until Sep 29th, 2025.

Cell Death Mechanism	Search Key for PubMed/MEDLINE	Search Key for SCOPUS	Search Key for Web of Science	Results for PubMed/ MEDLINE	Results for SCOPUS	Results for Web of Science	Results Removing Duplicates
**Inflammation**	("icariin") AND ("Neuro*") AND ("inflammation"[Mesh] OR "inflammation"[tiab])	"icariin" AND "Neuro*" AND "Oxidative Stress"	(TS="icariin") AND (TS="Neuro*") AND (TS="inflammation")	49	134	71	179
**Oxidative Stress**	("icariin") AND ("Neuro*") AND ("Oxidative Stress"[Mesh] OR "oxidative stress"[tiab])	"icariin" AND "Neuro*" AND "inflammation"	(TS="icariin") AND (TS="Neuro*") AND (TS="oxidative stress")	42	130	99	197
**Apoptosis**	("icariin") AND ("Neuro*") AND ("Apoptosis"[Mesh] OR "Apoptosis"[tiab])	"icariin" AND "Neuro*" AND "Apoptosis"	(TS="icariin") AND (TS="Neuro*") AND (TS="apoptosis")	76	155	114	229
**Necrosis**	"icariin" AND "Neuro*" AND ("Necrosis"[Mesh] OR "Necrosis"[tiab])	"icariin" AND "Neuro*" AND "Necrosis"	(TS="icariin") AND (TS="Neuro*") AND (TS="necrosis")	10	82	23	99
**Autophagy**	("icariin") AND ("Neuro*") AND ("Autophagy"[Mesh] OR "Autophagy"[tiab])	"icariin" AND "Neuro*" AND "Autophagy"	(TS="icariin") AND (TS="Neuro*") AND (TS="autophagy")	12	34	18	46
**Ferroptosis**	("icariin") AND ("Neuro*") AND ("Ferroptosis"[Mesh] OR "Ferroptosis"[tiab])	"icariin" AND "Neuro*" AND "Ferroptosis"	(TS="icariin") AND (TS="Neuro*") AND (TS="ferroptosis")	3	10	5	13
**Excitotoxicity**	("Icariin") AND ("neuro*") AND ("Excitotoxicity")	"Icariin" and "neuro*" and "Excitotoxicity"	(TS="icariin") AND (TS="Neuro*") AND (TS="excitotoxicity")	4	17	23	35
**Cancer**	("icariin") AND ("Neuro*") AND (("cancer"[tiab]) OR ("tumor"[tiab]))	"icariin" AND "Neuro*" AND ("cancer" OR "tumor")	(TS="icariin") AND (TS="Neuro*") AND ((TS="cancer") OR (TS="tumor"))	36	125	47	160

Source: Authors
